# IQGAP1 promotes chronic pain by regulating the trafficking and sensitization of TRPA1 channels

**DOI:** 10.1093/brain/awac462

**Published:** 2022-12-07

**Authors:** Shakil Khan, Pabitra Hriday Patra, Hannah Somerfield, Hattaya Benya-Aphikul, Manoj Upadhya, Xuming Zhang

**Affiliations:** School of Health & Life Sciences, Aston University, Birmingham B4 7ET, UK; School of Health & Life Sciences, Aston University, Birmingham B4 7ET, UK; School of Health & Life Sciences, Aston University, Birmingham B4 7ET, UK; School of Life Sciences, University of Warwick, Coventry CV4 7AL, UK; School of Health & Life Sciences, Aston University, Birmingham B4 7ET, UK; School of Health & Life Sciences, Aston University, Birmingham B4 7ET, UK; School of Life Sciences, University of Warwick, Coventry CV4 7AL, UK

**Keywords:** TRPA1, IQGAP1, protein trafficking, inflammatory pain, neuropathic pain, calcium signalling

## Abstract

TRPA1 channels have been implicated in mechanical and cold hypersensitivity in chronic pain. But how TRPA1 mediates this process is unclear. Here we show that IQ motif containing GTPase activating protein 1 is responsible using a combination of biochemical, molecular, Ca^2+^ imaging and behavioural approaches. TRPA1 and IQ motif containing GTPase activating protein 1 bind to each other and are highly colocalized in sensory dorsal root ganglia neurons in mice. The expression of IQ motif containing GTPase activating protein 1 but not TRPA1 is increased in chronic inflammatory and neuropathic pain. However, TRPA1 undergoes increased trafficking to the membrane of dorsal root ganglia neurons catalysed by the small GTPase Cdc42 associated with IQ motif containing GTPase activating protein 1, leading to functional sensitization of the channel. Activation of protein kinase A is also sufficient to evoke TRPA1 trafficking and sensitization. All these responses are, however, completely prevented in the absence of IQ motif containing GTPase activating protein 1. Concordantly, deletion of IQ motif containing GTPase activating protein 1 markedly reduces mechanical and cold hypersensitivity in chronic inflammatory and neuropathic pain in mice. IQ motif containing GTPase activating protein 1 thus promotes chronic pain by coupling the trafficking and signalling machineries to TRPA1 channels.

## Teaser

Khan *et al.* study the mechanisms by which TRPA1 channels contribute to mechanical and cold hypersensitivity. They show that the scaffold protein IQGAP1 is an essential part of this process, binding to TRPA1 channels and promoting their trafficking and functional sensitization in sensory neurons.

## Introduction

Chronic pain is a global health challenge causing enormous social and economic burden. It can be either nociceptive or neuropathic.^1^ Both involve peripheral sensitization through which the responsiveness of peripheral nociceptors is enhanced leading to pain hypersensitivity.^2–4^ This process critically depends on transduction channels, such as transient receptor potential (TRP) channels, on primary afferent fibres specialized for converting noxious stimuli into electrical signals.^3^ A classic example is sensitization of the heat-sensitive TRPV1 channels during inflammation leading to heat hyperalgesia.^5,6^ In contrast to heat hyperalgesia, the mechanisms of mechanical and cold hyperalgesia are less understood.

TRPA1 is another important nociceptive channel responsible for detection of diverse noxious stimuli. It is activated by a range of irritating chemicals, inflammatory agents and metabolites.^7^ Consistently, TRPA1 participates in chemical nociception in animals.^7–9^ In addition to chemoreception, TRPA1 has also been proposed as a sensor for mechanical and cold stimuli under physiological conditions,^8,10–17^ although it is highly controversial.^9,18,19^ Nevertheless, there is a growing consensus that TRPA1 is involved in mechanical and cold hypersensitivity in different types of chronic pain under pathological conditions.^20–33^ A key question is how does TRPA1 mediate apparent responses to mechanical and cold stimuli in pathological but not in physiological conditions? A tempting idea is that TRPA1 is sensitized under pathological conditions so that weak mechanical and cold stimuli can now activate TRPA1 to an appreciable level mediating pain hypersensitivity but otherwise evoke a marginal effect. However, this idea was not supported by a previous study.^34^ On the other hand, it was reported that inflammatory messengers sensitize TRPA1 and induce TRPA1 trafficking *in vitro.*^35–40^ But it remains elusive whether increased trafficking and sensitization of TRPA1 plays a role in mechanical and cold hypersensitivity *in vivo*.

Furthermore, the mechanisms of TRPA1 trafficking and sensitization are far from clear. Protein kinase A (PKA) and phospholipase C (PLC) signalling pathways have been implicated in the sensitization of TRPA1 induced by inflammatory agents.^33,35,36^ Further evidence suggests that PKA sensitizes TRPA1 through phosphorylation of the channel.^33,41,42^ However, PKA also promoted the trafficking of TRPA1 in dorsal root ganglia (DRG) neurons.^37^ This triggered the question of whether PKA sensitizes TRPA1 through enhancing the gating or trafficking of TRPA1 channels. Adding to the complexity, another study reported that sensitization of TRPA1 depends on interaction with TRPV1 channels.^43^

Interestingly, we have identified IQGAP1 as a novel binding partner of TRPA1 channels in sensory DRG neurons (see next). IQGAP1 is a large multi-domain scaffold protein interacting with numerous proteins such as Rho family of GTPases (Cdc42 and Rac1), ERK1/2, actin-cytoskeleton proteins and several transmembrane receptors.^44,45^ It serves as a hub of multiple signalling pathways mediating diverse cellular processes such as actin-cytoskeleton dynamics, cell adhesion/signalling, vesicle transport and protein trafficking,^45,46^ and has been extensively studied in cancer development and metastasis.^47^ However, whether IQGAP1 plays a role in pain signalling is not known.

In this research, we addressed these questions. We demonstrate that increased TRPA1 trafficking and sensitization in DRG neurons underlie mechanical and cold hypersensitivity in both chronic inflammatory and neuropathic pain. These processes critically depend on IQGAP1 that binds to TRPA1, because deletion of IQGAP1 not only abolished the trafficking and sensitization of TRPA1 channels in DRG neurons *in vitro*, but also markedly reduced mechanical and cold hypersensitivity in animals *in vivo*. Mechanistically, inflammatory signalling such as PKA and Ca^2+^ increases the binding of TRPA1 to IQGAP1, which then carries active Cdc42 anchored on IQGAP1 in proximity to TRPA1 catalysing the trafficking process of the channel. This study therefore revealed a macro chronic pain signalling complex formed between TRPA1, IQGAP1 and its associated signalling partners facilitating chronic pain by coupling signalling messengers and trafficking mechanisms to a nociceptive channel. We conclude that IQGAP1 is a critical player in chronic pain through regulating the trafficking and sensitization of TRPA1 channels.

## Materials and methods

### Animals

IQGAP1^−/−^ mice in a 129 background were obtained from Dr David Sacks (Department of Laboratory Medicine, National Institutes of Health) under the Material Transfer Agreement. Animals were maintained and housed in a 12 h light/dark cycle with food and water *ad libitum*. All experimental procedures were approved by the Animal Welfare and Ethical Review Body and complied with UK Home Office regulations and the Animal Scientific Procedures Act 1986 in the UK. Adult mice of both sexes aged between 8 and 16 weeks were used and randomly assigned to experimental groups.

### Animal models

To induce inflammatory pain, 20 μl of Complete Freund’s Adjuvant (CFA, Merck) was injected into the hind paws of mice. For neuropathic pain, we performed spared nerve injury (SNI) on mice as described by others.^48,49^ Briefly, under anaesthesia with isoflurane, incisions were made to expose the trifuracation of the sciatic nerve. The branches of common peroneal nerve and tibial nerves were ligated with sutures followed by distal transection. Muscle and skin in the wound were closed with sutures.

### Molecular biology

cDNA constructs coding for TRP channels including TRPA1-V5-His, TRPM8-V5-His and TRPV1-V5-His were generated as described previously.^6,50^ pCDNA3-Myc-IQGAP1 and GFP-tagged dominant-negative (DN) Cdc42 in pcDNA3 vector were purchased from Addgene. N-IQGAP1 (1M-Q905), C-IQGAP1 (L906-K1657) and C1-IQGAP1 (V1361-K1657) with a Flag tag at the N terminus were all polymerase chain reaction (PCR) amplified and subcloned into pCDNA3 vector. IQ motif (L700-Q905) deleted IQGAP1 (ΔCaM-IQGAP1), Cdc42 binding region (M1054-K1077) deleted IQGAP1 (ΔMK24-IQGAP1) and exocyst-binding region (V1361-Y1563) deleted IQGAP1 (ΔExo-IQGAP1) were generated using Quick-Change mutagenesis kit (Agilent). IQGAP1 shRNA 5′-GATCCGTGCCATGGATGAGATTGGAGAAGCTTGTCCAATCTCATCCATGGCA TTTTTTGGAAGC-3′ was cloned in pGSU6 vector. All the mutations were verified by gene sequencing.

### Behavioural assays

Animals were acclimatized to the testing environment for at least 3 h before behavioural assays. We then conducted hot plate, Von Frey, Hargreaves tests and acetone evaporation assay on the animals.

### Cell culture and transfection

Human embryonic kidney 293 (HEK293) cells were cultured and transfected as described previously.^51^ DRG was then dissociated and cultured as described previously with minor modifiations.^51^ Briefly, DRG was cultured on coverslips coated with poly-L-lysine (100 μg/ml). Nerve growth factor was not added to the culture to prevent unexpected effects. For imaging DRG neurons isolated from mice with inflammatory pain and neuropathic pain, only lumbar DRG (L3-L5) from the contralateral and ipsilateral sides were isolated. Dissociated DRG neurons were plated in droplets on the coverslips coated with poly-L-lysine and immediately used for calcium imaging within 5 h after isolation.

### Ca^2+^ imaging

DRG neurons on coverslips were loaded with Fura-2AM (ThermoFisher) at 37°C for 20 min. Coverslips were then mounted in a perfusion chamber on a Nikon inverted microscope connected to an automated perfusion system (Warner Instruments) through which drugs were delivered to the cells. The coverslip in the chamber was continuously perfused with Hanks’ balanced salt solution containing (in mM) 140 NaCl, 4 KCl, 10 HEPES, 1.8 CaCl_2_, 1 MgCl_2_, 5 glucose (pH 7.4). Fura-2AM loaded cells were exposed alternately for 50 ms to a 340 and 380 nm LED illuminator (Cairn Research UK). Emission was collected every 2 s at 510 nm using a sCMOS camera (Photometrics). The ratio of emission signals of 340 and 380 nm were calculated as an index of [Ca^2+^]*_i_*. A 10% increase in fluorescence ratio over baseline was considered as a response. We quantified the percentage of responding neurons by calculating the ratio of the number of responding neurons evoked by low dose allyl isothiocyanate (AITC) to the total number of TRPA1^+^ DRG neurons elicited by saturating dose AITC.

### Membrane protein detection and western blotting

Membrane proteins were labelled using biotinylation assay as described previously.^50^ We also isolated membrane protein using Mem-PER Plus Membrane Protein Extraction Kit (ThermoFisher) from DRG and sciatic nerves in accordance with instructions with mild modifications. Briefly, lumbar DRG were washed with 200 μl of wash buffer after isolation. They were then added to 200 μl of permeabilization buffer and homogenized using a motor-driven homogenizer followed by incubation at 4°C for 10 min. The cell suspension was centrifuged at 16 000 rpm for 15 min at 4°C. The cell pellet was next resuspended in the solubilization buffer containing protease inhibitors and homogenized. The cell solution was then incubated at 4°C for 30 min with constant mixing before centrifugation at 16 000 rpm for 15 min at 4°C. The supernatant was then used for western blotting as described before. Membrane TRPA1 was detected using anti-TRPA1 antibody (Alomone).

### Pull-down assay and co-immunoprecipitation

A nickel bead pull-down assay was used to detect interaction between TRPA1 and IQGAP1 in HEK293 cells expressing TRPA1-V5–6 × histidine and IQGAP1. It was performed as described previously.^52^ IQGAP1 and TRPA1 were detected using anti-IQGAP1(Santa Cruz) and anti-V5 (ThermoFisher), respectively.

To identify unknown proteins binding to TRPA1, nickel beads purified proteins from HEK293 cells expressing TRPA1-V5-6 × histdine were separated on 7.5% sodium dodecyl sulphate–polyacrylamide gel electrophoresis gel and then fixed and stained with silver staining kit (Sigma) in accordance with the manufacturer’s instructions. Protein bands of interest were excised and processed for liquid chromatography–tandem mass spectrometry (LC–MS/MS) analysis.

A GST pull-down assay was conducted as described previously.^52,53^ Bound proteins were detected using anti-IQGAP1 and/or -Flag (Merck). Co-immunoprecipitation was used to detect TRPA1-IQGAP1 interaction in DRG neurons and binding of HA-CaM to IQGAP1 in HEK293 cells. It was performed as described previously.^53^

### Immunohistochemistry

Mice were transcardially perfused with PBS and 4% paraformaldehyde (PFA). Lumbar DRG (L4-L5), sciatic nerves and skin were then isolated and post-fixed in 4% PFA followed by cryopreservation in 30% sucrose. Tissues were embedded in OCT medium (Tissue-Tek) and sectioned in a cryostat at 12 μm thickness. DRG sections were placed onto poly-lysine-coated slides for immunohistochemistry. DRG tissue sections were first blocked in 5% Donkey serum plus 0.03% Triton X-100 at room temperature for 30 min. They were then incubated with primary antibodies [mouse anti-IQGAP1 (Santa Cruz), rabbit anti-TRPA1 (Alomone), rabbit anti-CGRP (Merck) and rabbit anti-TRPV1 (Santa Cruz)] at 4°C overnight. After washing in PBS, DRG sections were next incubated with fluorescence-conjugated secondary antibodies [Alexa Fluor 594 conjugated donkey anti-mouse, Alexa Fluor 488 conjugated chicken anti-rabbit and FITC-conjugated Isolectin B4 (Merck)] at room temperature for 2 h. After a thorough wash, DRG slides were sealed by coverslips with Mowoil solution.

### Immunocytochemistry and live labelling

Immunocytochemistry in DRG neurons was performed as described previously.^6^ Membrane TRPA1 in DRG neurons was also live labelled as described.^37^ Briefly, DRG neurons were incubated with anti-TRPA1 directed against an extracellular epitope of TRPA1 at 37°C for 10 min. To label cytoplasmic IQGAP1, neurons were fixed in 4% PFA and permeabilized in 0.1% Triton X-100 followed by incubation with anti-IQGAP1 primary antibody and corresponding secondary antibody as described before.

### Quantitative PCR with reverse transcription

Lumbar DRG was rapidly isolated 7 days after CFA injection and SNI surgery. Total RNA was extracted using TRIzol reagent (Invitrogen) and reverse transcribed to cDNA using SuperScript^TM^ II reverse transcriptase (Invitrogen) in accordance with the manufacturer’s instructions. Quantitative PCR reactions were prepared by mixing cDNA with SYBR green master mix reagents (Applied Biosystems) and primer pairs in a 384-well plate and then amplified in a LightCycler 480 (Roche). All gene expression was normalized against GAPDH and analysed using the ΔΔCt method.

### Electrophysiology

Whole-cell electrophysiology were performed at room temperature as described previously.^51^ Small-diameter DRG neurons were recorded with patch pipettes fabricated from thin-walled glass capillary Pipette solution contains (in mM): 140 KCl, 2.0 MgCl_2_, 5 EGTA and 10 HEPES, pH 7.4 with KOH. Neurons were perfused with an extracellular solution consisting of (in mM): 140 NaCl, 4 KCl, 10 HEPES, 1 MgCl2, 5 EGTA and 5 glucose, pH 7.4 with NaOH. Series resistance was 80% compensated. Signals were analogue filtered at 1 kHz using a low-pass Bessel filter of the amplifier and digitized using Digidata 1440A (Molecular Devices).

### Statistics

All data are mean ± SEM. Significance between groups was determined using Student’s *t*-test or one- or two-way ANOVA followed by Bonferroni *post hoc* test. *P* < 0.05 was considered to be significant.

## Results

### Membrane TRPA1 is increased in dorsal root ganglia neurons in chronic inflammatory and neuropathic pain

**Figure 1:**
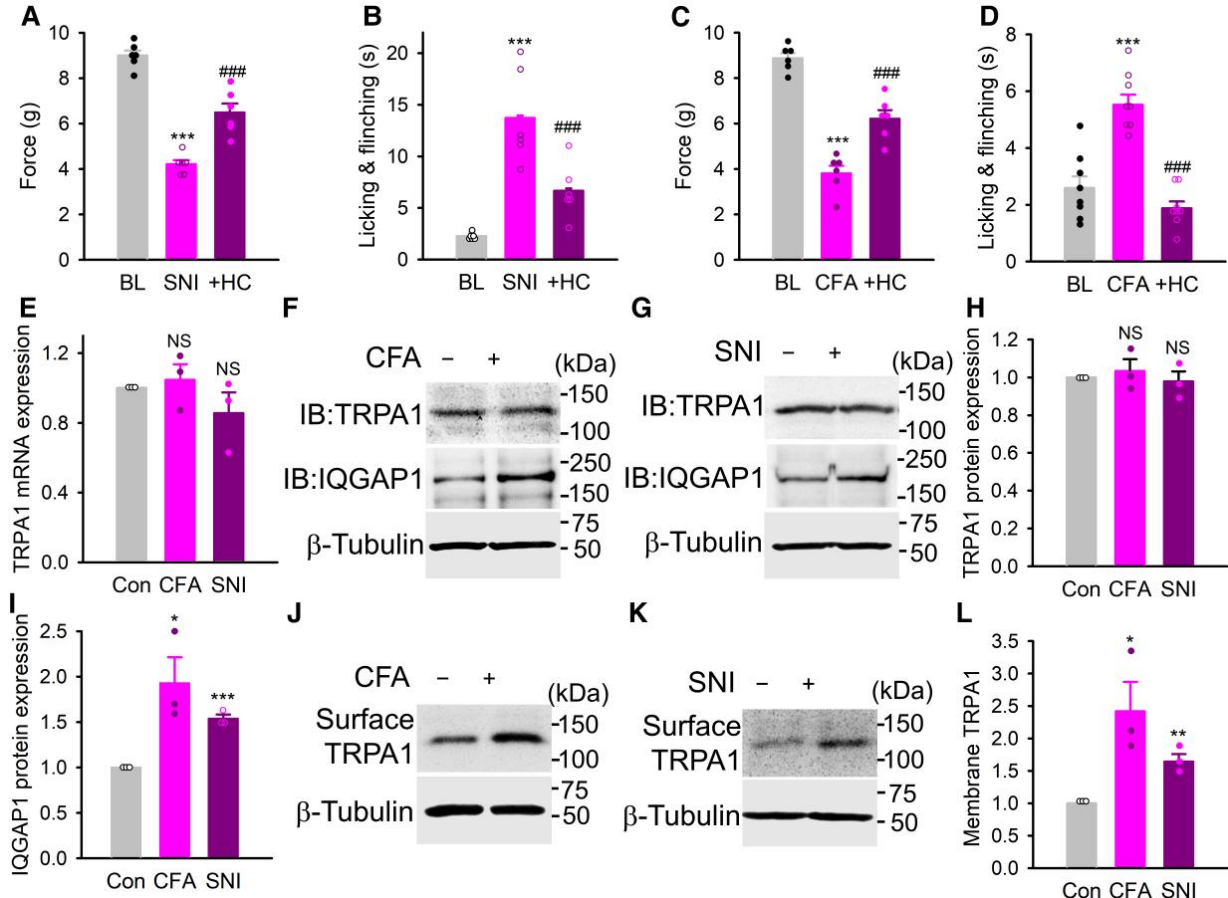
Increased TRPA1 trafficking is involved in mechanical and cold hypersensitivity in chronic pain. (**A** and **B**) Threshold mechanical force to paw withdrawal (**A**), time spent on nocifensive behaviours in acetone evaporation assay (**B**) in mice in baseline (BL), 7 days after SNI surgery or SNI mice injected (i.pl) with HC-030031 (1 mM, 10 μl). *n* = 6 per group. ***P < 0.001 compared to BL; ^###^*P* < 0.001 compared to SNI. (**C** and **D**) Threshold force in response to mechanical stimuli (**D**) (*n* = 6 per group) and duration of nocifensive responses in acetone evaporation assay in mice 7 days after CFA injection (i.pl) or with prior injection (i.pl) of HC-030031 (*n* = 8 per group). ***P < 0.001 compared to BL; ^###^*P* < 0.001 compared to CFA. (**E**) Normalized TRPA1 mRNA expression in lumbar DRG from CFA and SNI mice relative to control. *n* = 3, NS = not significant. (**F** and **G**) Expression of TRPA1 and IQGAP1 in lumbar DRG from mice 7 days after CFA injection (**F**) or SNI injury (**G**). Blots were stripped and redetected with anti-β-tubulin (*bottom*). (**H** and **I**) Summary of normalized expression of TRPA1 (**H**) and IQGAP1 (**I**) from experiments similar to those in **F** and **G**. *n* = 3, *P < 0.05; ***P < 0.001. (**J** and **K**) Surface TRPA1 expression in lumbar DRG from mice 7 days after CFA injection (**J**) or SNI surgery (**K**). Blots stripped and reprobed with anti-tubulin (*bottom*). (**L**) Normalized membrane expression of TRPA1 from similar experiments to those in **J** and **K**. *n* = 3. *P < 0.05; ***P < 0.001.

To establish the role of TRPA1 in mechanical and cold hypersensitivity in neuropathic pain, we generated an SNI model in mice and then assessed their sensitivity to mechanical and cold stimuli. The mice developed mechanical allodynia 7 days after surgery (Fig. 1A), as evidenced by reduced threshold mechanical force. The nerve injured mice also spent much longer time on nocifensive behaviours in response to cooling caused by acetone (Fig. 1B), indicating cold allodynia. Mechanical and cold allodynia were both markedly reduced after blocking TRPA1 by administrating the TRPA1 antagonist HC-030031 (Fig. 1A and B). Note that HC-030031 had no effect on the basal mechanical and cold sensitivity (Supplementary Fig. 1A and B). Similar effects were also observed when HC-030031 was administered to the mice at Day 14 of post-SNI surgery (Supplementary Fig. 1C and D). We also generated chronic inflammatory pain model by injection of CFA into the hindpaws of mice. As expected, mechanical and cold hypersensitivity became prominent 7 days after CFA injection (Fig. 1C and D). A previous blockade of TRPA1 by intraplantar injection of HC-030031 similarly prevented hypersensitive responses to mechanical and cold stimuli (Fig. 1C and D). These experiments suggest enhanced function of TRPA1 channels on sensory nerve endings underlies mechanical and cold hypersensitivity in chronic pain, although other mechanisms may also be involved.

It has been suggested that upregulated gene expression of TRPA1 is a cause of enhanced TRPA1 function.^24,26,28,54^ However, we found that neither TRPA1 mRNA nor protein was altered in lumbar DRG (L3-L5) isolated from mice at Day 7 of post-CFA and post-SNI when pain hypersensitivity was fully established (Fig. 1E and H). Instead, TRPA1 on the membrane of DRG neurons was significantly increased in both pain models (Fig. 1J and L), suggesting that increased TRPA1 trafficking to the membrane of DRG neurons underlies enhanced function of TRPA1 channels in chronic pain.

### IQGAP1 is a binding partner of TRPA1

**Figure 2:**
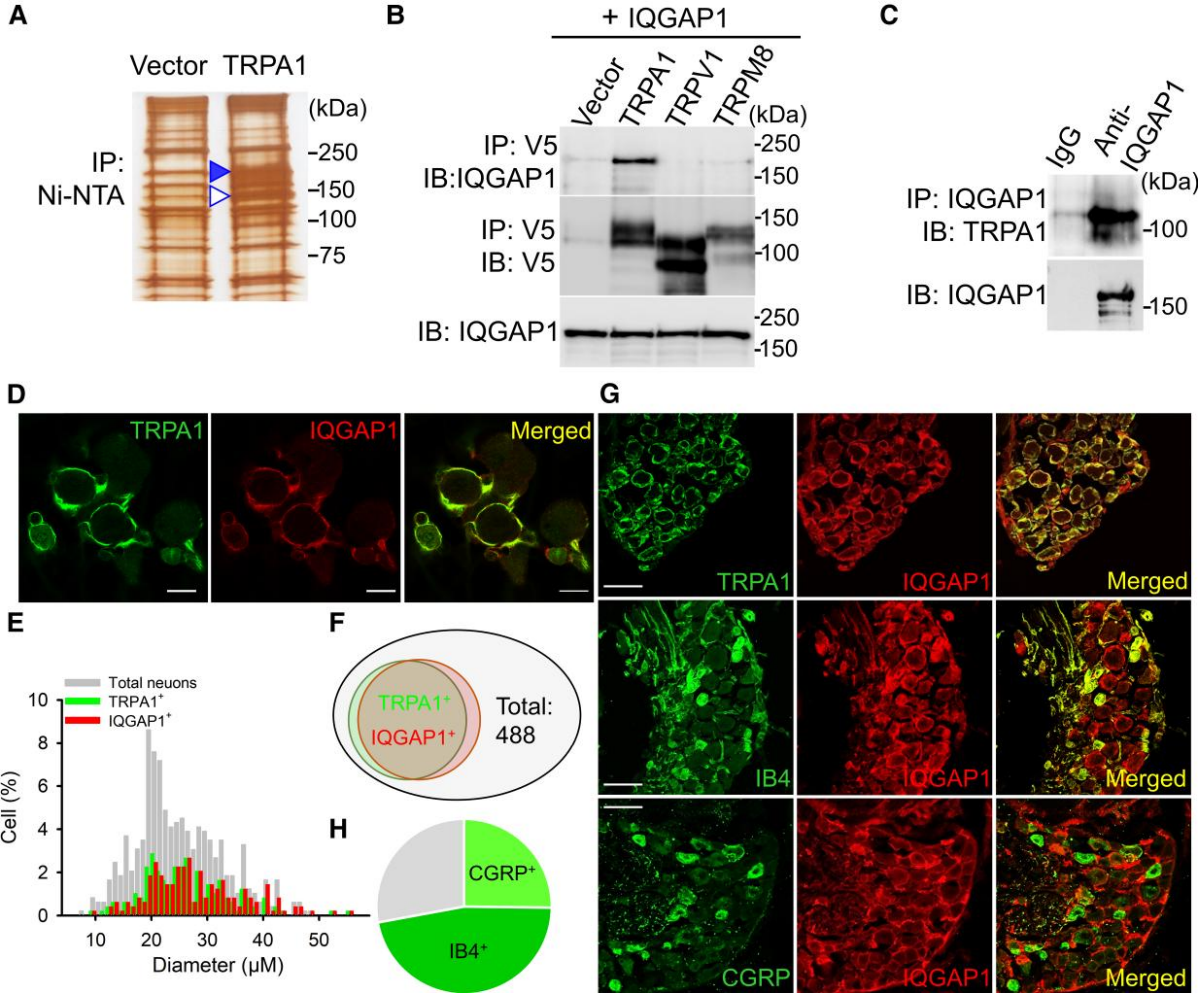
**TRPA1 binds to IQGAP1 and is colocalized with IQGAP1 in DRG neurons.** (**A**) Silver staining of proteins from HEK293 cell lysate containing TRPA1-His (6×) after purification by nickel beads. An open arrow indicates TRPA1, a solid arrow indicates unknow co-purified proteins. (**B**) IQGAP1 co-precipitates with TRPA1, but not with other TRP channels in co-immunoprecipitation using HEK293 cells transfected with cDNA constructs as indicated (*top*). The blot was stripped and reprobed with anti-V5 (*middle*). IQGAP1 expression is comparable in all groups (*bottom*). (**C**) Coprecipitation of TRPA1 with IQGAP1 in DRG neurons. (**D**) Colocalization of TRPA1 with IQGAP1 in DRG neurons. Scale bars = 20 μm. (**E**) Histogram distribution of TRPA1^+^ and IQGAP1^+^ DRG neurons as a function of cell diameter. *n*_total_ = 488, *n*_TRPA1_^+^ = 172, *n*_IQGAP1_^+^ = 179. (**F**) Venn diagram shows the relationship of TRPA1^+^ and IQGAP1^+^ neurons relative to all neurons (*n*_total_ = 488). (**G**) Co-expression of IQGAP1 with TRPA1, IB4 and CGRP in lumbar DRG. Scale bars = 50 μm. (**H**) Pie diagram depicts the proportion of CGRP^+^ (25.2 ± 2.7%, *n* = 11) and IB4^+^ (46.9 ± 4.8%, *n* = 9) DRG neurons within IQGAP1^+^ DRG neurons.

To ascertain the mechanisms of TRPA1 trafficking, we set out to identify the protein that binds to TRPA1 responsible for the trafficking of the channel. As TRPA1 trafficking can be recapitulated in HEK293 cells,^37^ we used HEK293 cells to express TRPA1 tagged with V5 and 6× histidine in the C terminus. We then purified TRPA1 from HEK293 cell lysate using nickel beads. Co-purified proteins were resolved on sodium dodecyl sulphate–polyacrylamide gel electrophoresis gel followed by silver staining. Interestingly, a prominent unknown protein band of ∼190 kDa was found to be co-purified with TRPA1 (Fig. 2A). The unknown band was then excised and subjected to LC–MS/MS analysis generating a list of protein candidates (Supplementary Table 1). Among them, IQGAP1 has a top score suggesting that IQGAP1 is a binding partner of TRPA1 channels.

We validated the binding of TRPA1 to IQGAP1 in both HEK293 cells expressing TRPA1 and IQGAP1 and in native DRG neurons using co-immunoprecipitation (Fig. 2B and C). Notably, IQGAP1 binds specifically to TRPA1 and does not bind to other TRP channels including TRPV1 and TRPM8 (Fig. 2B). Analysis of co-expression of two proteins in cultured DRG neurons revealed that 35.5% (173 out of 488) of DRG neurons expressed TRPA1 and 36.7% (179 out of 488) of DRG neurons expressed IQGAP1 (Fig. 2D). Notably, IQGAP1 was localized specifically in the juxtamembrane of DRG neurons and highly colocalized with TRPA1 (Fig. 2D). In fact, TRPA1^+^ and IQGAP1^+^ DRG neurons largely overlap with 93.6% (162 out of 173) of TRPA1^+^ DRG neurons co-expressing IQGAP1 and 90.5% (162 out of 179) of IQGAP1^+^ DRG neurons co-expressing TRPA1 (Fig. 2E and F). High co-expression and colocalization between TRPA1 and IQGAP1 were also evident on examination of lumbar DRG using immunohistochemistry (Fig. 2G). Interestingly, their co-expression was also found in sciatic nerve and skin tissues (Supplementary Fig. 2).

Further neurochemical analysis revealed that 49.2% (130 out of 264) of IQGAP1^+^ neurons were positive for IB4 and 73.0% (130 out of 178) of IB4^+^ neurons expressed IQGAP1. IQGAP1 was also partially co-expressed with CGRP with 21.9% (87 out of 398) of IQGAP1^+^ neurons expressing CGRP and 64.4% (87 out of 135) of CGRP^+^ neurons expressing IQGAP1 (Fig. 2G and H). Furthermore, IQGAP1 was co-expressed with TRPV1 with 83.5% (162 out of 194) of IQGAP1^+^ DRG neurons co-expressing TRPV1 (Supplementary Fig. 2). Therefore, IQGAP1 is expressed in both peptidergic and non-peptidergic DRG neurons, although it is mostly present in the non-peptidergic subpopulation, consistent with the previous finding that TRPA1 is mainly expressed in non-peptidergic DRG neurons.^55^

### Molecular delineation of TRPA1-IQGAP1 binding

**Figure 3:**
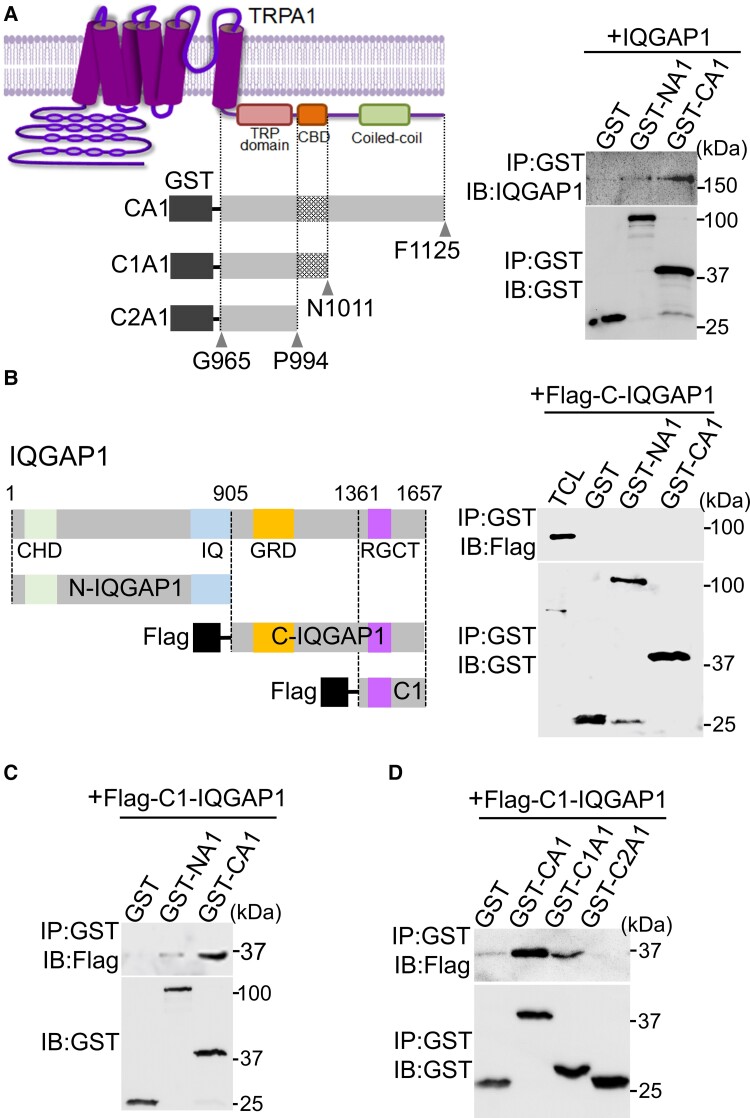
**Delineation of the mutual binding regions between TRPA1 and IQGAP1.** (**A**) IQGAP1 binds to the C terminus of TRPA1 in a GST pull-down assay (*right*). On the *left* is a schematic diagram showing the structure of TRPA1 and different C-terminal truncation fragments coupled to GST. (**B**) Schematic diagram depicting the different structural domains on IQGAP1 and constructed N- and C-IQGAP1 fragments with a Flag tag added to the N terminus. C-IQGAP1 does not bind to the cytoplasmic tails of TRPA1 in a GST pull-down assay (*right*). (**C**) The C terminus of TRPA1 binds to C1-IQGAP1 in a GST pull-down assay. (**D**) C1-IQGAP1 binds to the distal C terminus of TRPA1. All the blots were repeated at least three times.

We then characterized the mutual binding between TRPA1 and IQGAP1 as before.^53^ The cytoplasmic N and C termini of TRPA1 coupled to GST tag were first used to pull down IQGAP1. We found that IQGAP1 binds stronger to the C terminus than to the N terminus of TRPA1 (Fig. 3A). Complementarily, to determine TRPA1 binding regions on IQGAP1, the N- and C-terminal halves of IQGAP1 (i.e. N- and C-IQGAP1) were constructed to test their binding to the cytoplasmic tails of TRPA1. Interestingly, although C-IQGAP1 exhibited no detectable bindings to the cytoplasmic tails of TRPA1 (Fig. 3B), a further deletion of C-IQGAP1 containing solely the distal C-terminal portion (i.e. C1-IQGAP1) resulted in a prominent binding to the C terminus of TRPA1 (Fig. 3C), suggesting that the distal C terminus of IQGAP1 (1361–1657) is the major binding region for TRPA1. It also suggests that the N-terminal part of C-IQGAP1 contains an autoinhibitory domain masking TRPA1 binding region in the distal C terminus. Consistent with this idea, it has been reported previously that the GRD domain in the N-terminal portion of C-IQGAP1 binds to the RGCT domain in the distal C terminus mediating an autoinhibitory intramolecular interaction maintaining IQGAP1 in an inactive conformation.^56^ Binding of TRPA1 to the distal C terminus of IQGAP1 may disrupt this autoinhibitory intramolecular interaction leading to constitutive activation of IQGAP1.

To further delineate IQGAP1-binding regions in the C terminus of TRPA1, we generated two more truncated fragments. Deletion of distal 114 amino acids containing the coiled-coil domain from the C terminus of TRPA1 dramatically reduced the binding to C1-IQGAP1 (Fig. 3D). A further truncation of 17AA containing the CaM-binding region entirely abolished binding to C1-IQGAP1, showing that the distal C terminus of IQGAP1 binds primarily to the distal C terminus of TRPA1.

We then investigated the binding of N-IQGAP1 to the cytoplasmic tails of TRPA1. N-IQGAP1 did not bind to any C-terminal fragments of TRPA1 but exhibited a weak binding to the N terminus (Supplementary Fig. 3A and C). Further mapping studies revealed that N-IQGAP1 binds to the proximal N terminus of TRPA1 between M450 and C652 (Supplementary Fig. 3B and C).

Altogether, TRPA1-IQGAP1 binding is mainly mediated by their distal C terminus through a C–C interaction, although there is also a weak N–N interaction between their N termini.

### IQGAP1 promotes TRPA1 trafficking

**Figure 4:**
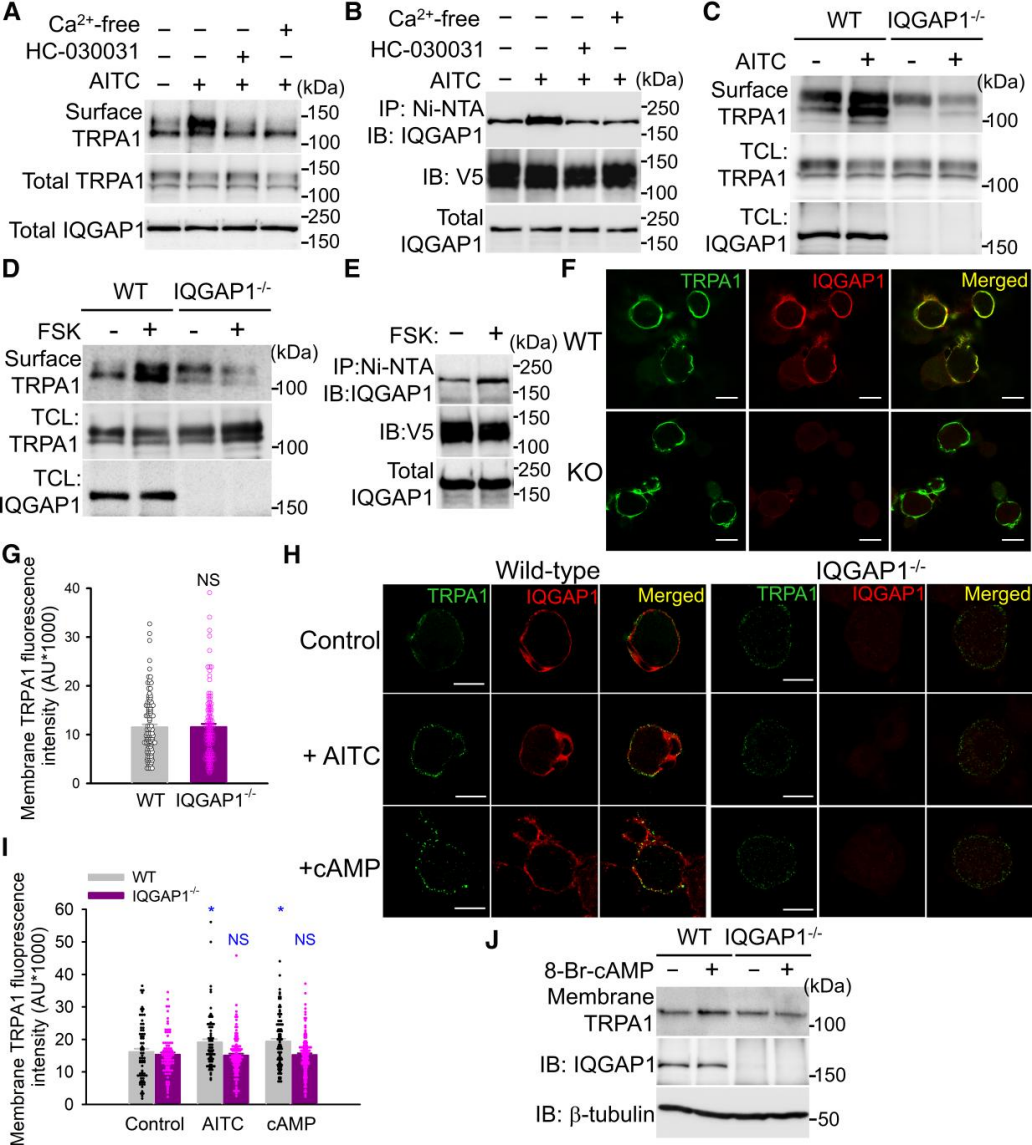
**IQGAP1 is essential to TRPA1 trafficking induced by Ca^2+^ and PKA.** (**A**) Biotinylation of membrane TRPA1 in HEK293 cells expressing TRPA1 treated with AITC (400 μM, 4 min) or in the presence of HC030031 (10 μM) or Ca^2+^-free solution containing 5 mM EGTA. Band densities in the top blot, relative to the first band, are: 1.0, 1.8, 0.7, 0.8. Total TRPA1 and IQGAP1 are similar across groups. (**B**) Binding of IQGAP1 to TRPA1 in a Ni-NTA pull-down assay in HEK293 cells transfected with TRPA1-V5-his (6×) activated by AITC (400 μM) or together with HC-030031 (10 μM) or in Ca^2+^-free condition. Relative band densities 1.0, 1.63, 0.83 and 0.82. (**C** and **D**) Biotinylation of membrane TRPA1 in WT and IQGAP1^−/−^ HEK293 cells expressing TRPA1 after activation by AITC (400 μM) (**C**) and forskolin (FSK) (50 μM, 10 min). Band densities in **C**: 1.0, 1.29, 0.62, 0.48. **D**: 1.0, 1.60, 1.17, 0.75. (**E**) FSK increases the interaction of TRPA1 with IQGAP1 in a Ni-NTA pull-down assay. TRPA1 and IQGAP1 expression are similar in both groups. Band densities 1.0, 1.71. (**F**) Examples images showing fluorescence of membrane TRPA1 and cytoplasmic IQGAP1 in WT and IQGAP1 knockout (KO) DRG neurons. (**G**) Summary of quantification of membrane TRPA1 signals in experiments similar to those in **F**. WT, *n* = 110; IQGAP1^−/−^, *n* = 108. NS = not significant. (**H**) Live labelling membrane TRPA1 in DRG neurons treated with AITC (100 μM, 4 min) or 8-Br-cAMP (50 μM, 10 min) in WT and IQGAP1 lacking DRG neurons. (**I**) Summary of quantification of membrane TRPA1 fluorescence intensity from experiments similar to those in **H**. *n* = 71∼121 per group. *P < 0.05; NS = not significant. (**J**) Fractionation of membrane TRPA1 in DRG neurons (*top*) with or without treatment by 8-Br-cAMP (50 μM, 10 min). The blots were stripped and reprobed with anti-IQGAP1 (*middle*) and anti-β-tubulin (*bottom*). Band densities 1.0, 1.20, 0.96, 0.90. All the blots were repeated at least three times.

IQGAP1 has been implicated in actin-cytoskeleton remoulding and protein traffic in different cellular systems.^46,57^ We then wondered whether IQGAP1 regulates TRPA1 trafficking. We over-expressed TRPA1 in HEK293 cells expressing endogenous IQGAP1 and then labelled membrane TRPA1 using a membrane protein biotinylation assay. Activation of TRPA1 with AITC pronouncedly enhanced surface TRPA1 (Fig. 4A). This process depends on Ca^2+^ influx through TRPA1 channels because TRPA1 trafficking was abolished by either blocking TRPA1 with HC-030031 or by chelating extracellular Ca^2+^ ([Ca^2+^]_e_) with EGTA (Fig. 4A), consistent with the previous report.^37^ Interestingly, AITC treatment also concomitantly increased the binding of IQGAP1 to TRPA1 (Fig. 4B). This event was also blocked by either HC-030031 or by removing [Ca^2+^]*_e_* (Fig. 4B), suggesting that enhanced binding of TRPA1 to IQGAP1 underlies increased TRPA1 trafficking. To confirm this idea, we performed similar experiments in IQGAP1-lacking HEK293 cells in which endogenous IQGAP1 is genetically ablated^58^ and found that increased TRPA1 trafficking elicited by AITC was eliminated (Fig. 4C), demonstrating that IQGAP1 is essential to TRPA1 trafficking induced by Ca^2+^ signalling.

In addition to Ca^2+^, activation of PKA has also been shown to increase TRPA1 trafficking and sensitize TRPA1,^37,41,42^ although the underlying mechanisms remain unknown. To determine whether IQGAP1 is also involved in PKA-induced TRPA1 trafficking, similar experiments were also performed in wild-type (WT) and IQGAP1-lacking HEK293 cells. As anticipated, activation of PKA with forskolin markedly increased membrane TRPA1 (Fig. 4D). Concurrently, TRPA1-IQGAP1 binding was also increased (Fig. 4E). However, this effect was abolished in IQGAP1 knockout cells (Fig. 4D). To avoid any unwanted compensatory mechanisms caused by knocking out IQGAP1, we also conducted experiments in cells in which IQGAP1 is knocked down with shRNA and found the similar results (Supplementary Fig. 4A). These results demonstrate that IQGAP1 is also essential to TRPA1 trafficking induced by PKA signalling. It is noteworthy that the basal membrane TRPA1 was not significantly affected by either ablating IQGAP1 or overexpressing IQGAP1 (Fig. 4C and D, Supplementary Fig. 4B and C). Collectively, these data suggest that IQGAP1 binding is critical to increased TRPA1 trafficking elicited by both Ca^2+^ and PKA signalling, albeit without effect on the basal membrane TRPA1.

To determine whether IQGAP1 is also critical to TRPA1 trafficking in DRG neurons, we labelled membrane TRPA1 using an antibody recognizing extracellular epitope of the channel under non-permeabilized conditions followed by permeabilizing the cells to detect cytosolic IQGAP1. Membrane TRPA1 and cytosolic IQGAP1 were localized in close proximity (Fig. 4F), supporting physical interaction between two proteins. We found that the basal membrane TRPA1 was indistinguishable between WT and IQGAP1 lacking DRG neurons (Fig. 4F and G), confirming that IQGAP1 does not influence the basal membrane TRPA1.

To investigate the role of IQGAP1 in TRPA1 trafficking evoked by Ca^2+^ and PKA signalling in DRG neurons, we labelled membrane TRPA1 in live DRG neurons after stimulation with AITC and 8-Bromo-cAMP and then fixed and permeabilized the cells to label intracellular IQGAP1. Both treatments significantly increased membrane TRPA1 in WT DRG neurons (Fig. 4H). However, these effects were abolished in DRG neurons from IQGAP1-KO mice (Fig. 4H and I). The finding was further confirmed by fractionation of membrane TRPA1 protein in DRG neurons (Fig. 4J). Collectively, these data demonstrate that IQGAP1 is crucial to TRPA1 trafficking triggered by both Ca^2+^ and PKA signalling.

### Mechanisms of TRPA1 trafficking

**Figure 5:**
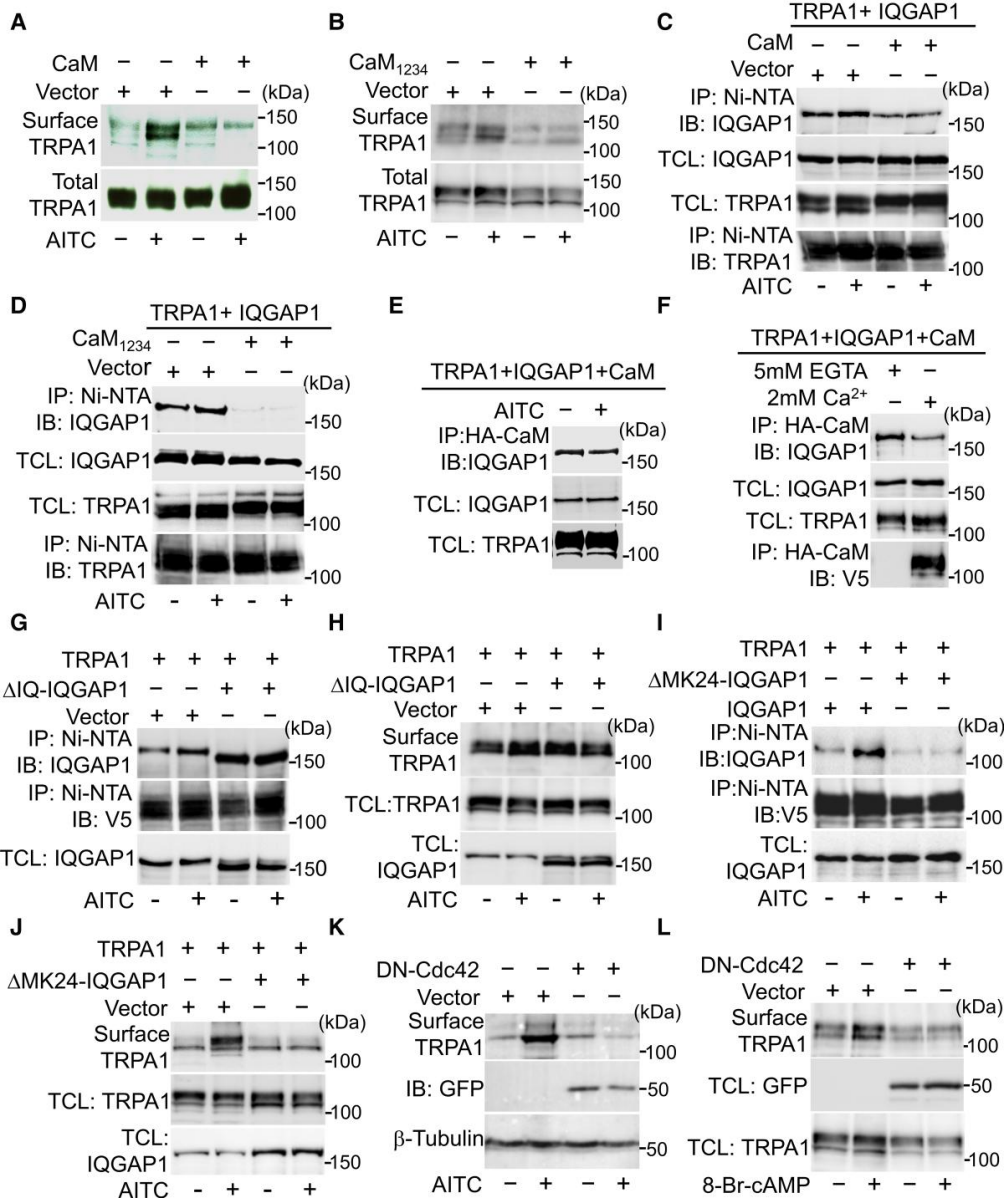
**Ca^2+^/CaM and Cdc42 regulate the trafficking of TRPA1 channels.** (**A** and **B**) Biotinylation of membrane TRPA1 in HEK293 cells transfected with TRPA1 and CaM or CaM_1234_ as indicated after activation by AITC (400 μM). Relative band densities in the top blot in **A**: 1.0, 2.81, 1.29, 0.91; **B**: 1.0, 1.25, 0.64, 0.75. Total TRPA1 are similar in all groups. (**C** and **D**) Overexpression of CaM (**A**) and CaM_1234_ (**B**) prevents binding of IQGAP1 to TRPA1 under basal and stimulated conditions by AITC (400 μM, 4 min) in a Ni-NTA pull-down assay. Band densities in **C**: 1.0, 1.24, 0.67, 0.72. **D**: 1.0, 1.18, 0.22, 0.20. TRPA1 and IQAGP1 expression in total cell lysate (TCL) are equivalents in all groups. (**E**) CaM binding to IQGAP1 is reduced in HEK293 cells expressing TRPA1, IQGAP1 and HA-CaM after activation by AITC in co-immunoprecipitation. Band densities: 1.0, 0.83. (**F**) CaM binds to less IQGAP1 in Ca^2+^ than in Ca^2+^-free (with 5 mM EGTA) revealed by co-immunoprecipitation. However, CaM binds to TRPA1 in Ca^2+^ but not in Ca^2+^-free. Band densities: 1.0, 0.65. (**G** and **H**) Binding of ΔIQ-IQGAP1 to TRPA1 is increased in a Ni-NTA pull-down assay (**G**) and prevents increases in membrane TRPA1 induced by AITC (400 μM) in a membrane protein biotinylation assay (**H**) in HEK293 cells transfected with cDNA as indicated. Band densities in **G**: 1.0, 1.37, 1.61, 1.64. **H**: 1.0, 1.25, 1.22, 1.08. (**I**, **J**) ΔMK24-IQGAP1 abolishes increased binding between TRPA1 and IQGAP1 in a Ni-NTA pull-down assay (**I**) and prevents TRPA1 trafficking induced by AITC (400 μM) in membrane protein biotinylation assay (**J**) in HEK293 cells transfected with cDNA as indicated. Band densities in **I**: 1.0, 2.04, 0.53, 0.56; **J**: 1.0, 1.96, 1.01, 0.99. (**K** and **L**) Co-expression of GFP-tagged DN-Cdc42 prevents increased membrane expression of TRPA1 induced by AITC (400 μM, 4 min) in a membrane protein fractionation assay (**K**) or by 8-Br-cAMP (50 μM, 10 min) in membrane protein biotinylation (**L**). Band densities in **K**: 1.0, 2.71, 1.06, 0.67; **L**: 1.0, 1.35, 0.71, 0.72. All the blots were repeated at least three times.

We next aimed to understand how IQGAP1 modulates TRPA1 trafficking. IQGAP1 regulates vesicle tethering, exocytosis and protein traffic by scaffolding diverse signalling molecules using different domains.^46,59^ Of note, IQGAP1 contains four tandem IQ motifs responsible for binding to Ca^2+^-loaded CaM (Ca^2+^-CaM) and Ca^2+^-free CaM (ApoCaM) mediating Ca^2+^-dependent regulation of IQGAP1 (Fig. 3B).^60,61^ We have previously found that the C terminus of TRPA1 contains a noncanonical CaM-binding domain binding to Ca^2+^-CaM but not to ApoCaM, mediating rapid Ca^2+^-dependent regulation of TRPA1.^53^ In experiments for determining the role of CaM in Ca^2+^-induced TRPA1 trafficking, we found that AITC-induced TRPA1 trafficking was prevented by overexpression of both CaM and Ca^2+^-free CaM_1234_ (ApoCaM), although CaM and CaM_1234_ had no effect on the basal membrane TRPA1 (Fig. 5A and B, Supplementary Fig. 4D). Correspondingly, CaM and CaM_1234_ also prevented increased binding between TRPA1 and IQGAP1 elicited by AITC (Fig. 5C and D). These experiments suggest that Ca^2+^ regulates TRPA1 trafficking and IQGAP1 binding through CaM anchored on IQGAP1. It has been suggested that CaM acts as a central negative regulator of IQGAP1 by preventing other partner proteins from binding to IQGAP1.^62^ Indeed, overexpression of CaM or CaM_1234_ caused a robust reduction in the binding of IQGAP1 to TRPA1 even under the basal condition (Fig. 5C and D, Supplementary Fig. 4E). We then reasoned that Ca^2+^-loaded CaM after TRPA1 opening should exhibit reduced binding to IQGAP1 so that the inhibitory effect of CaM on IQGAP1 is relieved mediating increased TRPA1 binding and trafficking.

Consistent with this idea, activation of TRPA1 with AITC reduced CaM binding to IQGAP1 (Fig. 5E). Furthermore, CaM exhibited much less binding to IQGAP1 in the presence of Ca^2+^ than without Ca^2+^ (Fig. 5F). In contrast, when Ca^2+^ was present, IQGAP1 bound more to TRPA1 and TRPA1 bound more to CaM (Fig. 5F and Supplementary Fig. 4F), consistent with our previous findings.^63^ Increased binding of TRPA1 to Ca^2+^-CaM may sequester a pool of Ca^2+^-CaM contributing to reduced binding of Ca^2+^-CaM to IQGAP1. To further determine the role of Ca^2+^/CaM in Ca^2+^-induced TRPA1 trafficking, we constructed ΔIQ-IQGAP1 in which all four IQ motifs are deleted from IQGAP1.^64^ As anticipated, there was a massive reduction in the binding of CaM to ΔIQ-IQGAP1 (Supplementary Fig. 4G). In contrast, the binding of ΔIQ-IQGAP1 to TRPA1 was markedly increased even under the basal condition, but there were no further increases in TRPA1 binding after activation of TRPA1 with AITC (Fig. 5G), which is expected because the ability of ΔIQ-IQGAP1 to sense Ca^2+^ is ablated.^64^ Consistent with the binding effect, ΔIQ-IQGAP1 co-expression markedly increased the basal membrane TRPA1 but abrogated increased membrane trafficking of TRPA1 evoked by AITC (Fig. 5H), further supporting the idea that IQGAP1 binding is critical to TRPA1 trafficking and that Ca^2+^ regulation of TRPA1 trafficking is mediated by CaM on IQGAP1 but not through CaM on TRPA1. Taken together, these results indicate a mechanistic model by which Ca^2+^ regulates TRPA1 trafficking.

We next wondered how IQGAP1 promotes TRPA1 trafficking. IQGAP1 regulates protein traffic by integrating at least two different trafficking mechanisms: first, IQGAP1 binds to Cdc42 through the GRD domain stabilizing Cdc42 in a GTP-bound active conformation, facilitating actin-cytoskeleton dynamics and vesicle trafficking^46^; second, IQGAP1 binds to the exocyst complex promoting vesicle tethering and exocytosis.^59,65^ To determine which mechanism is critical, we selectively ablated the binding domains on IQGAP1 for Cdc42 and exocyst complex, respectively, as described.^65,66^ Deletion of the binding domain for exocyst did not affect TRPA1 trafficking induced by AITC (Supplementary Fig. 4H). However, deletion of only 24 amino acids in the GRD domain of IQGAP1 (ΔMK24-IQGAP1) was sufficient to abolish increased TRPA1 binding and trafficking caused by AITC (Fig. 5I and J), suggesting that Cdc42 anchored on IQGAP1 is crucial to TRPA1 trafficking. To verify this idea, we co-expressed TRPA1 with dominant-negative Cdc42 (DN-Cdc42). Interestingly, DN-Cdc42 completely prevented increased TRPA1 trafficking induced by both AITC and 8-Bromo-cAMP (Fig. 5K and L), suggesting that Cdc42 anchored on IQGAP1 is active and that active Cdc42 is a key mechanism responsible for catalysing TRPA1 trafficking.

### IQGAP1 mediates TRPA1 sensitization caused by PKA

**Figure 6:**
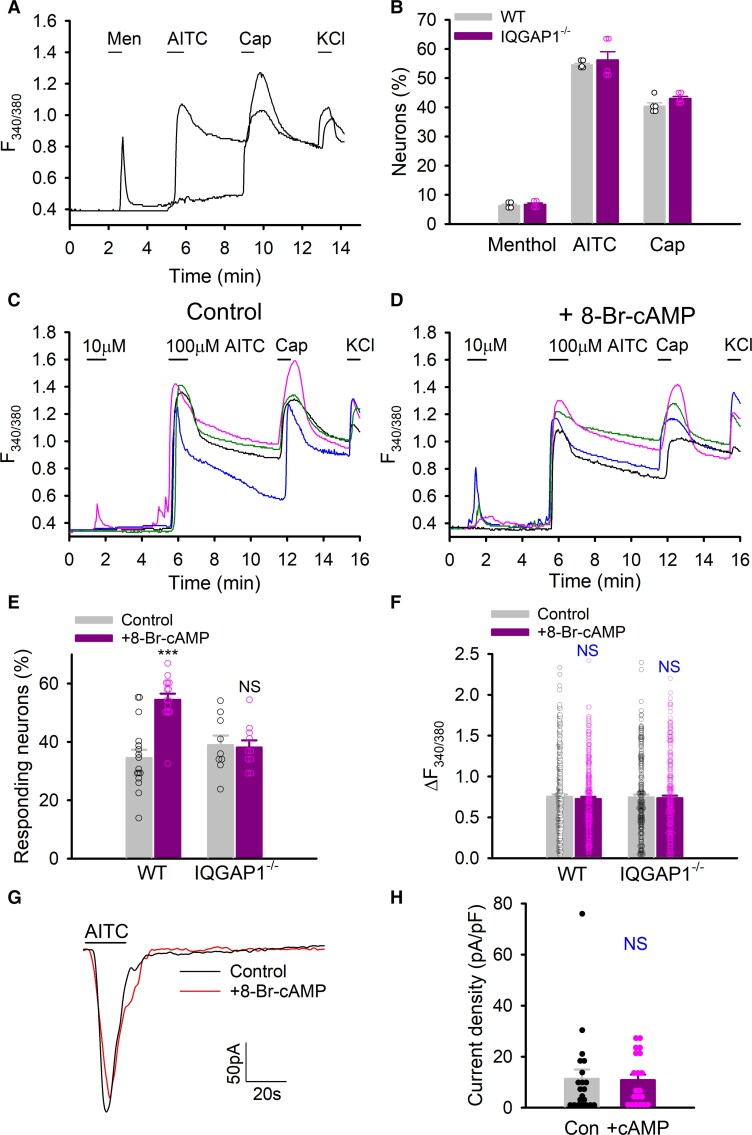
**Sensitization of TRPA1 by PKA depends on IQGAP1 in DRG neurons.** (**A**) Example Ca^2+^ responses in DRG neuros stimulated by menthol (Men, 100 μM), AITC (100 μM), capsaicin (Cap, 1 μM) and KCl (50 mM). (**B**) Summary of percentage of responding neurons to different stimuli in C. *n* = 4–6 mice per group, *n*_cell_ = 297–642 per experiment. (**C** and **D**) Example Ca^2+^ responses in DRG neurons in control (**C**) and pretreated with 8-Br-cAMP (50 μM, 10 min) (**D**) elicited by AITC, capsaicin (1 μM) and KCl. (**E**) Summary of percentage of neurons responding to 10 μM AITC from similar experiments to those in **E** and **F**. *n* = 9–16. *n*_cell total_ = 233–406 per group. ***P < 0.001; NS = not significant. (**F**) Summary of peak Ca^2+^ amplitudes in response to 100 μM AITC from experiments similar to those in **C** and **D**. WT Con, *n*_cell_ = 319; WT + cAMP, *n*_cell_ = 286; KO Con, *n*_cell_ = 209; KO + cAMP, *n*_cell_ = 246. NS, not significant. (**G**) Example peak inward currents in DRG neurons activated by AITC (100 μM, 20 s) with or without 8-Br-cAMP (50 μM). (**H**) Summary of current density from experiments similar to those in **G**. Control, *n* = 21; 8-Br-cAMP, *n* = 20. NS = not significant.

We then examined the role of IQGAP1 in the regulation of TRPA1 function in DRG neurons. Whole-cell inward peak current density activated by AITC and capsaicin in small-diameter DRG neurons were indistinguishable between WT and IQGAP1 knockout mice (Supplementary Fig. 5A). Calcium imaging also shows that the percentage of responding neurons evoked by the agonists for TRPM8, TRPV1 and TRPA1 channels were not significantly different between WT and IQGAP1-lacking DRG neurons (Fig. 6A and B). These results demonstrate that IQGAP1 does not affect the basal responses of TRPM8, TRPA1 and TRPV1 channels.

We next investigated whether IQGAP1 mediates PKA-induced sensitization of TRPA1 using Ca^2+^ imaging. Activation of TRPA1 causes rises in [Ca^2+^]*_i_*, which then rapidly potentiates or desensitizes TRPA1 depending on the levels of [Ca^2+^]*_i_*.^53^ TRPA1 therefore undergoes variable rapid autoregulation of its own function once activated, making it challenging to preclude the interference of Ca^2+^-dependent autoregulation of TRPA1 in TRPA1 sensitization caused by other mechanisms. A second challenge is variable sensitivity of TRPA1 channels across different subpopulations of DRG neurons.^43^ Of note, *de novo* sensitization of DRG neurons was found to be a major contributor to cold allodynia,^67,68^ but often neglected in the traditional detection method. To address these issues, we devised a two-dose protocol to probe TRPA1 sensitization. Neurons were first stimulated with a low subthreshold dose of AITC (10 μM) that evokes mild Ca^2+^ responses followed by a second saturating dose (100 μM) to maximally activate all the channels on the neuron membrane. We then measured TRPA1 sensitization by quantifying the percentage of neurons responding to the low dose (Fig. 6C). Meanwhile, the second saturating peak responses were used to estimate all TRPA1^+^ neurons and the total TRPA1 channels trafficking to the membrane. The two-dose protocol thus provides a dual approach to probing high and low sensitive TRPA1 channels, respectively, allowing for a better discrimination of neurons with variable sensitivities.

With this protocol, we found that prior activation of PKA with 8-Br-cAMP significantly increased the percentage of DRG neurons responding to low dose AITC (Fig. 6C and E). Notably, most AITC-sensitive DRG neurons also responded to capsaicin, thus co-expressing TRPV1. The sensitizing effect of PKA was, however, completely abolished in DRG neurons deficient for IQGAP1 (Fig. 6E). These data demonstrate that IQGAP1 is essential for TRPA1 sensitization induced by PKA congruent with the requirement of IQGAP1 for PKA-induced trafficking of TRPA1 (Fig. 4H and J). They also suggest that PKA-induced sensitization of TRPA1 is mainly mediated by TRPA1 trafficking. However, peak Ca^2+^ amplitudes caused by the second saturating dose after 8-Br-cAMP treatment were not significantly different from those in control (Fig. 6F). To preclude the possible indirect effects of the first low dose AITC on TRPA1 peak amplitudes evoked by the second saturating dose, neurons were also first stimulated with a saturating dose AITC. No significant differences were observed either (Supplementary Fig. 5B and C). We also used electrophysiology to directly record TRPA1 currents and did not find significant difference in TRPA1 currents after PKA activation (Fig. 6G and H). Presumably, PKA mainly sensitizes a subset of initially silent DRG neurons mediating *de novo* sensitization of TRPA1, an event known to be critical to cold allodynia.^67^ Peak responses in these *de novo* sensitized TRPA1 would be small and thus contribute little to the overall maximal peak responses in TRPA1^+^ DRG neurons.

### Chronic inflammation and nerve injury sensitize TRPA1 depending on IQGAP1

**Figure 7:**
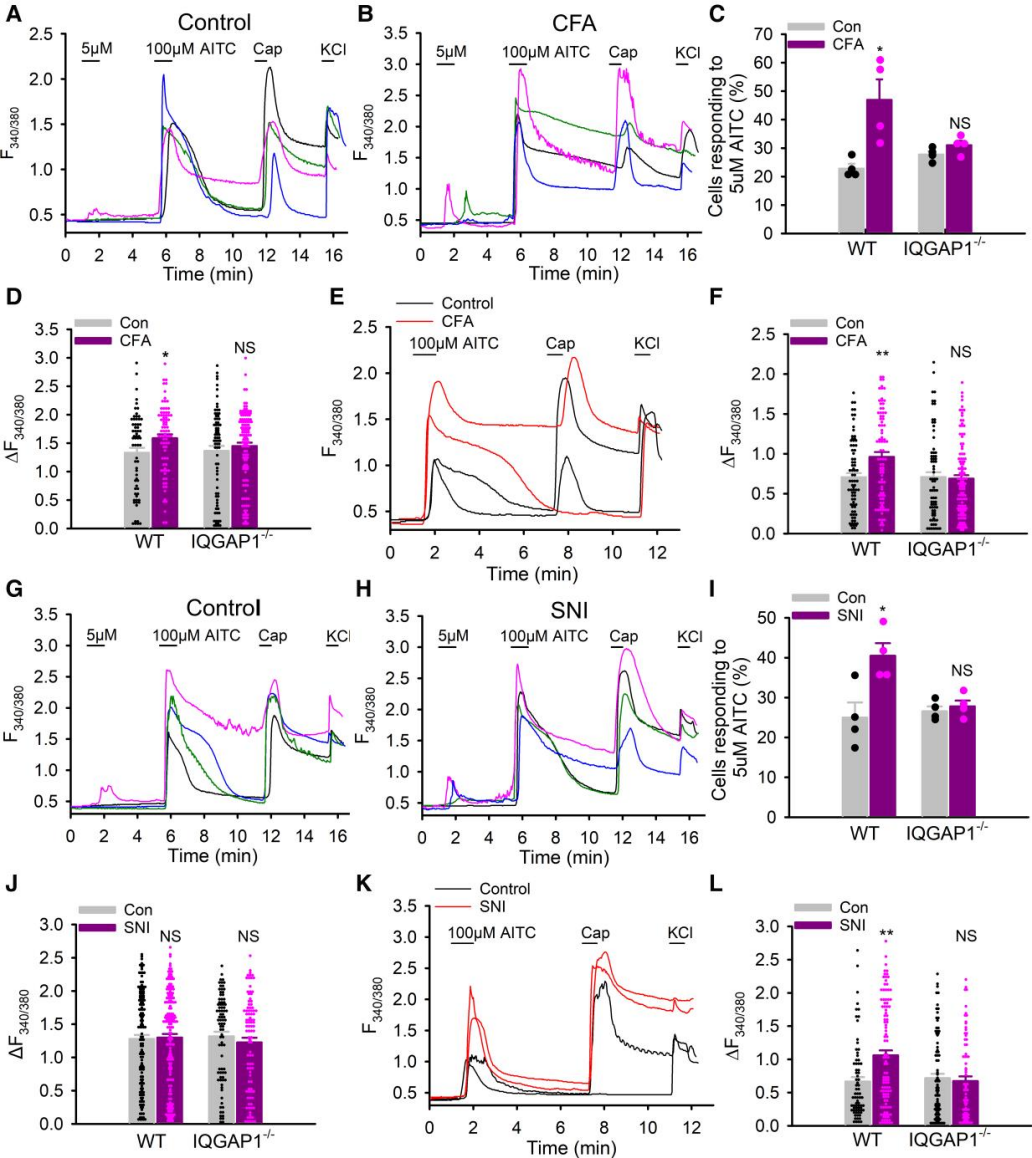
**IQGAP1 is crucial to TRPA1 sensitization in chronic inflammatory and neuropathic pain**. (**A** and **B**) Example Ca^2+^ responses in the contralateral (Control, **A**) and ipsilateral (CFA, **B**) lumbar DRG neurons from WT and IQGAP1^−/−^ mice 7 days after CFA injection. (**C**) Summary of percentage of DRG neurons responding to 5 μM AITC in control and CFA-evoked inflammatory pain. *n* = 4, *n*_cell_ = 105–178 per group. (**D**) Summary of peak Ca^2+^ amplitude in DRG neurons in response to 100 μM AITC from similar experiments to those in **A** and **B**. WT Con, *n*_cell_ = 66; WT CFA, *n*_cell_ = 81; KO Con, *n*_cell_ = 78; KO CFA, *n*_cell_ = 134. (**E**) Example Ca^2+^ responses in the contralateral and ipsilateral DRG neurons acutely dissociated from CFA-injected (i.pl) mice. (**F**) Summary of peak Ca^2+^ amplitudes in response to 100 μM AITC from experiments similar to those in **E**. *n* = 4–5 per group, WT Con, *n*_cell_ = 78; WT CFA, *n*_cell_ = 86; KO Con, *n*_cell_ = 76; KO CFA, *n*_cell_ = 113. (**G** and **H**) Typical Ca^2+^ responses in the contralateral (**G**, Con) and ipsilateral (**H**, SNI) lumbar DRG neurons isolated from WT and IQGAP1^−/−^ mice 7 days after SNI injury. (**I**) Summary of percentage of DRG neurons responding to 5 μM AITC in control and SNI mice. *n* = 4. *n*_cell_ = 117–185. (**J**) Collective results of peak Ca^2+^ amplitude in response to 100 μM AITC from experiments similar to those in **G** and **H**. *n* = 4. WT Con, *n*_cell_ = 156; WT SNI, *n*_cell_ = 132; KO Con, *n*_cell_ = 206; KO SNI, *n*_cell_ = 128. (**K**) Typical Ca^2+^ responses in the contralateral and ipsilateral (SNI) DRG neurons from SNI mice. (**L**) Summary of peak Ca^2+^ amplitude evoked by 100 μM AITC from experiments similar to those in **K**. WT Con, *n*_cell_ = 74; WT SNI, *n*_cell_ = 107; KO Con, *n*_cell_ = 88; KO SNI, *n*_cell_ = 76. *P < 0.05, **P < 0.01; NS = not significant.

To determine whether increased TRPA1 trafficking in chronic inflammatory and neuropathic pain in Fig. 1J and L causes corresponding functional sensitization of TRPA1, we acutely isolated contralateral and ipsilateral lumbar DRG neurons from mice 7 days after CFA injection or nerve injury and then assessed TRPA1 responses using Ca^2+^ imaging. These acutely dissociated DRG neurons are more sensitive than cultured neurons probably due to different oxidative states of TRPA1 channels, and 5 μM AITC was therefore used as a low dose instead. We found that CFA injection significantly increased the percentage of neurons responding to low dose AITC (Fig. 7A and C). Furthermore, peak Ca^2+^ amplitudes evoked by the saturating dose were also significantly increased (Fig. 7B and D), supporting increased trafficking of TRPA1 channels to the membrane. This effect is in contrast with the lacking effect of PKA on the peak Ca^2+^ responses in cultured DRG neurons evoked by high-dose AITC (Fig. 6F). It is likely that CFA activates wholesale inflammatory signalling in addition to PKA leading to stronger TRPA1 sensitization. To rule out the indirect effects of the first TRPA1 responses on the second peak TRPA1 responses, DRG neurons were also first stimulated with a saturating dose AITC (Fig. 7E). In this case, the difference in peak Ca^2+^ amplitude was even more significant between the contralateral and ipsilateral DRG neurons (Fig. 7F). We then performed similar experiments using IQGAP1-lacking mice and found that deletion of IQGAP1 not only prevented increases in the percentage of TRPA1^+^ DRG neurons in response to low dose AITC, but also abolished enhancement in the peak Ca^2+^ amplitude elicited by the saturating dose AITC (Fig. 7C, D and F).

In SNI neuropathic pain, nerve injury also significantly increased the proportion of TRPA1^+^ DRG neurons responding to low dose AITC (Fig. 7G and I). However, peak Ca^2+^ responses evoked by the second saturating dose were not significantly different from those in control (Fig. 7J). But when neurons were first stimulated with a saturating dose without a prior application of low dose AITC, elicited peak TRPA1 responses were now significantly larger in the ipsilateral DRG neurons than those in control (Fig. 7K and L). All these effects were, however, eliminated in IQGAP1-lacking DRG neurons (Fig. 7I and L). These Ca^2+^ imaging data were further consolidated by whole-cell patch clamping used to directly record peak TRPA1 currents in acutely dissociated DRG neurons from CFA and SNI mice (Supplementary Fig. 6A, B and C). These results suggest that TRPA1 is sensitized in chronic inflammatory and neuropathic pain through increased channel trafficking and that IQGAP1 is essential to these processes. Note that most TRPA1^+^ DRG neurons also responded to capsaicin, consistent with the notion that TRPA1 is expressed in a subset of TRPV1^+^ DRG neurons.^69^

Our experiments also show that consecutive activation of TRPA1 does influence and alter later TRPA1 responses. The idea is also supported by the finding that peak TRPA1 responses evoked by the saturating dose AITC were constantly significantly smaller than those in neurons pretreated with low dose AITC even under control conditions (Supplementary Fig. 6D and E). The difference is probably due to rapid modulation of TRPA1 by the first evoked Ca^2+^ responses^53^ and/or modification of the redox state of TRPA1 channels by the first exposure to AITC. These data suggest that consecutive activation of TRPA1 for probing TRPA1 sensitization used previously by others could indirectly influence and even distort TRPA1 responses, and is therefore questionable.

### IQGAP1 contributes to chronic pain hypersensitivity through regulating TRPA1 trafficking and sensitization

**Figure 8:**
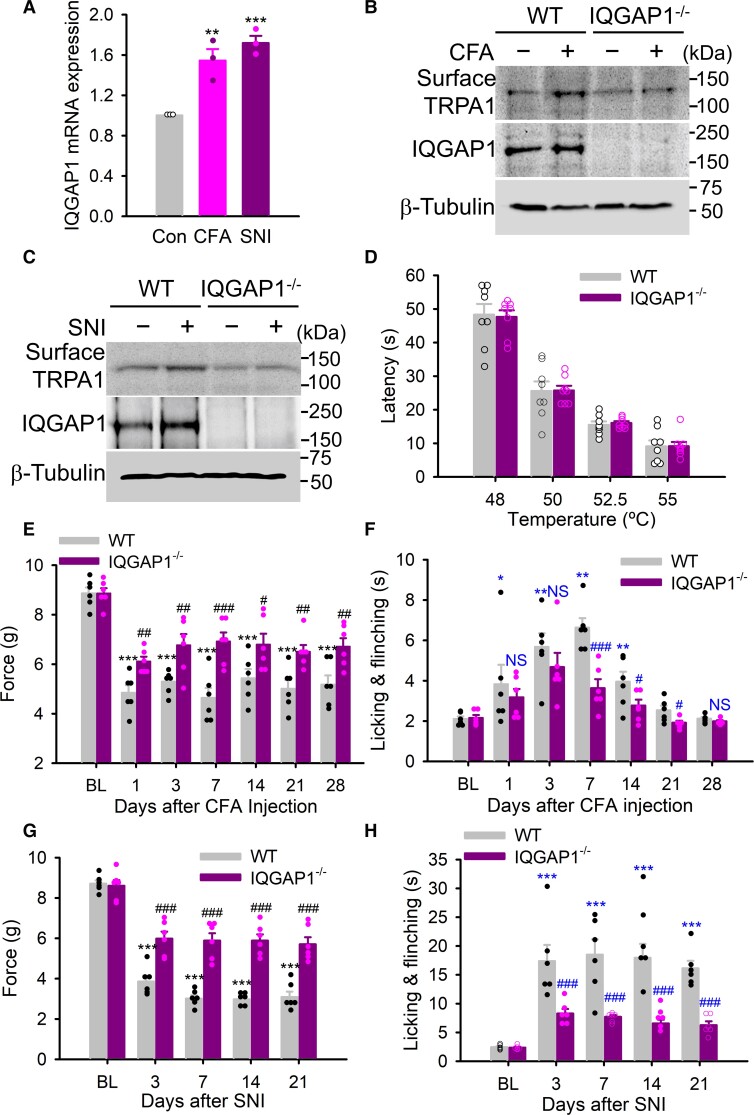
**IQGAP1 is critical to chronic inflammatory and neuropathic pain by promoting TRPA1 trafficking in DRG neurons.** (**A**) IQGAP1 expression in lumbar DRG neurons from mice 7 days after CFA injection and SNI injury. *n* = 3, *P < 0.05; **P < 0.01. (**B** and **C**) Surface TRPA1 is increased in lumbar DRG from mice 7 days after CFA (**B**) and SNI injury (**C**), but not from mice lacking IQGAP1. IQGAP1 protein is also increased in both cases. Note that TRPA1 and IQGAP1 were detected simultaneously using two antibodies. β-tubulin is loading control. Relative band densities in the top blot in **B**: 1.0, 1.34, 0.94, 1.0; **C**: 1.0, 1.23, 0.84, 0.88. (**D**) Summary of latency to nocifensive responses of WT and IQGAP1^−/−^ mice on hot plate at different temperatures. *n* = 8 per group. (**E**) Threshold mechanical force to paw withdrawal in WT and IQGAP1 knockout mice at different days after CFA injection. *n* = 6 per group. ***P < 0.001 compared to baseline (BL); ^#^*P* < 0.05; ^##^*P* < 0.01; ^###^*P* < 0.001 compared to WT. (**F**) Time spent on nocifensive responses of mice in acetone evaporation assay at different days after CFA injection. *n* = 6, *P < 0.05; **P < 0.01; ^#^*P* < 0.05; ^###^*P* < 0.001. (**G**) Threshold force to von Frey filament in WT and IQGAP1^−/−^ mice after SNI injury. *n* = 6. ***P < 0.001 compared to baseline (BL); ^###^*P* < 0.001 compared to WT. (**H**) Duration of licking and flinching behaviours in response to acetone application to the paws. *n* = 6. ***P < 0.001 compared to BL; ^###^*P* < 0.001 compared to WT.

We next wondered whether IQGAP1 sensitizes TRPA1 in chronic pain through regulating TRPA1 trafficking similar to those observed in cultured DRG neurons *in vitro* (Fig. 4H and J). Interestingly, we found that both IQGAP1 mRNA and protein were significantly increased in the ipsilateral DRG isolated from mice 7 days after CFA injection and nerve injury (Figs 1F, G, I and 8A), although no significant changes in either TRPA1 mRNA or protein were observed under the same conditions (Fig. 1F and H). Increased IQGAP1 was also found in the sciatic nerve (Supplementary Fig. 7A and B), suggesting that IQGAP1 is a critical regulator of chronic pain probably through modulating TRPA1 trafficking. To test this idea, we isolated lumbar DRG from mice at Day 7 of post-CFA and post-SNI followed by membrane protein fractionation. Consistent with the previous results (Fig. 1J and L), membrane TRPA1 in lumbar DRG was increased after CFA and SNI (Fig. 8B and C). However, all these effects were abolished in IQGAP1 deficient mice (Fig. 8B and C). Interestingly, similar effects were also observed in sciatic nerves isolated from these mice (Supplementary Fig. 7A and B). These data demonstrate that IQGAP1 promotes TRPA1 trafficking in both DRG neurons and sciatic nerves in chronic pain *in vivo.* The findings are also consistent with the essential role of IQGAP1 in mediating TRPA1 sensitization in chronic inflammatory and neuropathic pain (Fig. 7). Thus, IQGAP1 determines both the trafficking and sensitization of TRPA1 channels in chronic pain.

We finally determined whether increased TRPA1 trafficking and sensitization mediated by IQGAP1 contribute to mechanical and cold hypersensitivity using IQGAP1 knockout mice. IQGAP1^−/−^ mice exhibit normal body weight and normal basal sensitivity to thermal and mechanical stimuli without overt difference in gross appearance and behaviour. In a hot plate assay, these mice exhibited normal withdrawal responses compared to littermate controls (Fig. 8D). We next generated CFA-induced chronic inflammatory pain and monitor their pain behaviours. As expected, mechanical hypersensitivity was developed 1 day after CFA injection lasting for over 4 weeks. However, mechanical hypersensitivity was significantly blunted in IQGAP1 deficient mice for the entire study period (Fig. 8E). The mice also developed cold hypersensitivity from Day 1 of post-CFA lasting for 3 weeks (Fig. 8F). Cold hypersensitivity was also significantly reduced in IQAGP1-lacking mice between days 7 and 21 after CFA injection. These data demonstrate that IQGAP1 contributes to mechanical and cold hypersensitivity in chronic inflammatory pain.

The effect of IQGAP1 on neuropathic pain was also tested in mice with SNI. WT mice developed mechanical allodynia from day 3 of post-surgery lasting for over 3 weeks (Fig. 8G). They also developed robust cold allodynia, as indicated by pronounced increases in nociception behaviours in response to acetone cooling (Fig. 8H). Both mechanical and cold allodynia were markedly reduced in IQGAP1 knockout mice, albeit incompletely (Fig. 8G and H). However, cold allodynia was completely abolished when HC-030031 was administered to further block existing TRPA1 channels on the membrane in IQGAP1-KO mice (Supplementary Fig. 7C). Mechanical allodynia was also significantly further reduced close to the basal line level, although incompletely abrogated under the same conditions (Supplementary Fig. 7D), supporting that both newly inserted and existing membrane TRPA1 channels contribute to mechanical and cold allodynia, two classical symptoms of neuropathic pain. Altogether, these experiments show that IQGAP1 participates in chronic inflammatory and neuropathic pain by increasing the trafficking and function of TRPA1 channels.

## Discussion

TRPA1 has been implicated in mechanical and cold hypersensitivity in different types of chronic pain,^20–30,32,70,71^ although it remains controversial about whether TRPA1 is a direct sensor for mechanical and cold stimuli.^8,9,13–16,18,19^ In this research, we demonstrated an obligatory role of IQGAP1 in mechanical and cold hypersensitivity in chronic inflammatory and neuropathic pain through regulating the trafficking and sensitization of TRPA1 channels. We also elucidated the molecular mechanisms of TRPA1 trafficking mediated by IQGAP1 and found that IQGAP1 promotes TRPA1 trafficking via Cdc42 anchored on IQGAP1.

Nociceptor sensitization is a unique mechanism in pain transduction. It lowers the threshold for detection of noxious stimuli, as exemplified by TRPV1-mediated heat hyperalgesia,^5,6,50^ in which TRPV1 is sensitized so that warm temperatures can activate TRPV1 even though warmth is not a physiological TRPV1 activator. TRPA1 is another polymodal nociceptor.^7^ Although it is well established that TRPA1 is a chemoreceptor for many chemicals,^7,19^ activation of TRPA1 by mechanical and cold stimuli is variable, suggesting that mechanical and cold stimuli are at most weak activators for TRPA1. However, sensitized TRPA1 may enable the channel to transduce mechanical and cold stimuli effectively mediating mechanical and cold hypersensitivity in pathological conditions. Consistent with this idea, inflammation sensitized TRPA1-mediated firing responses of C-fibre to mechanical stimuli,^38^ and *de novo* sensitization of TRPA1 was suggested to be a mechanism of cold allodynia.^67^ Our research consolidated the important role of TRPA1 sensitization in mechanical and cold hypersensitivity in chronic pain. First, the sensitivity of TRPA1 in DRG neurons was increased in chronic inflammatory and neuropathic pain; second, blockade of TRPA1 reduced mechanical and cold hypersensitivity and third, prevention of TRPA1 sensitization by ablating IQGAP1 reduced mechanical and cold hypersensitivity.

However, TRPA1 sensitization was not found in a previous study in DRG neurons from mice with chronic constriction injury,^34^ although TRPA1 sensitization was consistently observed in CFA-induced inflammatory pain.^34,43^ The discrepancy may be caused by different protocols and neuropathic pain models (chronic constriction injury versus SNI model). A key posit of TRPA1 sensitization is the threshold for TRPA1 activation is lowered rendering TRPA1 more sensitive to weak stimuli. Furthermore, a subset of DRG neurons with silent sensitivity to mechanical and cold stimuli have been found to be important in mediating mechanical and cold hypersensitivity through *de novo* sensitization.^68,72^ Presumably, these neurons are susceptible to be regulated to gain *de novo* sensitivity. For these reasons, it is more meaningful to use weak stimuli to probe TRPA1 sensitization. We thus developed a two-dose protocol with low dose AITC used for isolating very sensitive and *de novo* sensitized neurons, while saturating dose AITC (100 μM) for triggering maximal peak responses reflecting TRPA1 trafficking and total TRPA1^+^ DRG neurons. The advantages of this protocol are therefore 2-fold. Our data obtained with this protocol endorse the idea that increased TRPA1 trafficking is a major mechanism of TRPA1 sensitization in DRG neurons leading to mechanical and cold hypersensitivity in chronic pain. A single higher dose AITC (50 μM) used in the previous study probably activated almost all TRPA1^+^ DRG neurons with little differentiation, masking the small subpopulation of sensitized TRPA1^+^ DRG neurons.^34^

The next key question is how TRPA1 is sensitized during chronic pain. A popular idea is that upregulated TRPA1 gene expression causes TRPA1 sensitization and pain hypersensitivity.^24,26,28,54^ However, we did not find any significant changes in either TRPA1 mRNA or protein in chronic inflammatory and neuropathic pain. It is largely consistent with others who found that TRPA1 mRNA was transiently increased at Day 1 and 3 after CFA injection but returned to the normal level afterwards.^26,34^ However, it is variable about TRPA1 expression under nerve injury conditions with some reporting a mild increase in TRPA1 mRNA,^24,26,54^ while others reporting a reduction in TRPA1 mRNA.^34^ The differences presumably involve different types of nerve injury models used. Of note, a recent study reported that there is no alteration in TRPA1 mRNA in DRG from mice with SNI,^73^ consistent with our findings. In our research, we examined both TRPA1 mRNA and protein and found no significant differences in all cases. Strikingly, in stark contrast to TRPA1, we found that IQGAP1 mRNA and protein were significantly increased in both CFA and SNI models, supporting that IQGAP1 is an important regulator of chronic pain and TRPA1 sensitization.

Sensitization of TRPA1 could be due to either enhanced gating or trafficking of the channel. Previous studies found that PKA sensitizes TRPA1 through phosphorylation of the channel,^33,41,42^ alluding to enhanced TRPA1 gating as an important mechanism of TRPA1 sensitization. However, our evidence supports that increased TRPA1 trafficking is responsible for TRPA1 sensitization with little involvement of channel gating: first, PKA also enhanced the binding of TRPA1 to IQGAP1 mediating increased TRPA1 trafficking (Fig. 4D and E); second, prevention of TRPA1 trafficking by deletion of IQGAP1 not only entirely abolished TRPA1 sensitization in DRG neurons induced by PKA (Fig. 6E) but also prevented TRPA1 sensitization in the settings of chronic inflammatory and neuropathic pain (Fig. 7) and third, PKA-induced sensitization of TRPA1 is much delayed typically occurring ∼5–10 min after treatment (Fig. 6D),^37,41,43^ consistent with latency required for protein traffic. In contrast, direct channel gating occurs much rapidly taking typically <1 min.

We went on to decipher the mechanisms of TRPA1 trafficking and found that enhanced binding of TRPA1 to IQGAP1 is a common mechanism underlying increased TRPA1 trafficking caused by PKA and Ca^2+^ signalling. Upregulated expression of IQGAP1 during chronic pain could further contribute to increased TRPA1-IQGAP1 binding and TRPA1 trafficking. Mechanistically, Cdc42 anchored on IQGAP1 was found critical to TRPA1 trafficking, supporting the model that IQGAP1 brings active Cdc42 adjacent to TRPA1 through IQGAP1-TRPA1 signalling complex catalysing TRPA1 trafficking. Cdc42 is a molecular switch known to promote actin-cytoskeleton remoulding and vesicle trafficking through interaction with various downstream effectors such as β-catenin, CLIP170, N-WASP and Arp2/3.^74^ Thus, IQGAP1 and associated multiple signalling messengers form a convergent pain signalling complex with TRPA1 mediating TRPA1 trafficking and sensitization induced by different inflammatory signalling. This signalling complex diversifies the regulatory mechanisms of TRPA1, providing a tantalizing solution to other unresolved questions in TRPA1 biology.

Interestingly, a previous study also reported that TRPA1 trafficking contributes to acute mechanical hypersensitivity induced by Wnt3 signalling depending on Rac1,^75^ belonging to the same family of small GTPases such as Cdc42. Increased TRPA1 trafficking has also been implicated in diabetic neuropathic pain.^76^ It will be interesting to investigate whether IQGAP1 is also critical to TRPA1 trafficking in these conditions. These studies highlight a broad role for TRPA1 trafficking and small GTPase in the regulation of chronic pain.

In summary, this research revealed the mechanisms of chronic pain hypersensitivity mediated by TRPA1 and the mechanisms of TRPA1 trafficking and sensitization, highlighting a central role for IQGAP1 in these processes. IQGAP1 and small GTPase may constitute an alternative route for therapeutic interventions for the treatment of chronic pain.

## Supplementary Material

awac462_Supplementary_Data

## Data Availability

The data used to support the conclusions in this paper are available from the corresponding author upon reasonable request.
