# Supplementary material for: IQGAP1 promotes chronic pain by regulating the trafficking and sensitization of TRPA1 channels
Source: Brain. 2022 Dec 7;146(6):2595–611. doi: 10.1093/brain/awac462 (PMC10232262; doi:10.1093/brain/awac462)

Supplementary materials and methods

Animals

IQGAP1$^{-/-}$ mice in 129 background were obtained from Dr. David Sacks (Department of Experiments Medicine, National Institute of Health) under the Material Transfer Agreement (MTA). Animals were maintained at Aston University and the University of Warwick and housed in a 12h light/dark cycle with food and water *ad libitum*. All experimental procedures were approved by the Animal Welfare and Ethical Review Body (AWREB) in both Universities and complied with UK Home Office regulations and the Animal Scientific Procedures Act 1986 in the UK. Adult mice of both sexes aged between 8-16 weeks were used and randomly assigned to experimental groups.

Animal models

To induce inflammatory pain, 20μl Complete Freund's Adjuvant (CFA, Merck) was injected into the hind paws of mice. For neuropathic pain, we performed spared nerve injury (SNI) on mice as described by others[48, 49]. Briefly, under anaesthesia with isoflurane, an incision was made to expose the biceps femoris (BMF) muscle on the thigh of the hind limb of mice. A blunt section was then made through BMF exposing the trifuracation of the sciatic nerve. The branches of common peroneal nerve and tibial nerves were ligated with sutures followed by distal transection and removing 2mm of the distal nerve stump. Muscle and skin in the wound were closed with sutures.

Molecular Biology

cDNA constructs coding for TRP channels including TRPA1-V5-His, TRPM8-V5-His and TRPV1-V5-His were generated as described previously[6, 50]. pCDNA3-Myc-IQGAP1 and GFP-tagged dominant-negative (DN) Cdc42 in pcDNA3 vector were purchased from Addgene. N-IQGAP1 (1M-Q905) was PCR amplified and subcloned into pCDNA3 vector via KpnI and XbaI. C-IQGAP1 (L906-K1657) and C1-IQGAP1 (V1361-K1657) was amplified using PCR with a Flag tag added to their N-terminus followed by subcloning into pCDNA3 vector via BamHI and EcoRI. IQ motif (L700-Q905) deleted IQGAP1 (ΔCaM-IQGAP1), Cdc42 binding

region (M1054-K1077) deleted IQGAP1 (ΔMK24-IQGAP1) and exocyst-binding region (V1361-Y1563) deleted IQGAP1 (ΔExo-IQGAP1) were generated using Quick-Change mutagenesis kit (Agilent). IQGAP1 shRNA 5'-GATCCGTGCCATGGATGAGATTGGAGAAGCTTGTCCAATCTCATCCATGGCA TTTTTTGGAAGC-3' was cloned in pGSU6 vector. All the mutations were verified by gene sequencing.

Behavioural assays

Animals were acclimatized to the testing environment for at least 3h prior to behavioural assays which were typically carried out in the middle of the day.

Hot plate: Mice were placed on a temperature-controlled surface with a transparent plexiglass cylinder restraining the animals (Ugo basile). Latency to nocifensive behaviours such as paw licking, flinching, lifts and jumping was determined.

Von Frey test: The mechanical sensitivity of the mice was assessed using a dynamic plantar aesthesiometer (Ugo Basile). Animals were placed on metal grid pane enclosed by a transparent box (9.6 x 9.6 x 14.5cm) and habituated for at least 30 min prior to experiment. To measure mechanical sensitivity, the plantar surface of mice was stimulated by a rigid von Frey filament with increasing force. The threshold force at which animals withdraw their paws was recorded. 2-3 trials were tested for each measurement with 10min gap between each trial.
Hargreaves test: the thermal sensitivity of mice was measured using a Hargreaves apparatus (Ugo Basile). After thorough habituation of mice on a glass frame plane enclosed by a plastic box, infrared heat was delivered from underneath of the paws. Time latency to paw withdrawal was then automatically recoded.

Acetone evaporation assay: cold sensitivity of animals was assessed using acetone evaporation assy. A drop of 50μl acetone was delivered to the plantar surface of the hindpaws of mice, time spent on licking, flinching and biting was recorded.

Cell culture and transfection

HEK293 cells were maintained in DMEM medium (4.5g/l glucose) supplemented with 10% FBS, 2mM L-glutamine ,100 units/ml penicillin and 100μg/ml streptomycin in a humidified incubator containing 5% CO2, as described previously[51]. Cells were transfected with TurboFect transfection reagents (Thermo Fisher Scientific) as described previously[51].

DRG was rapidly isolated from adult mice after sacrifice by cervical dislocation. DRG was then dissociated and cultured as described previously with minor modifiations[51]. Briefly, DRG was treated with Type IV collagenase (Worthington) at 37°C for 30min followed by trituration with a 23G needle. Cells were then filtered through a Falcon cell strainer (Corning) to remove debris and pelleted by centrifugation. DRG neurons were resuspended in DMEM medium (1g/l glucose) supplemented with L-glutamine (2mM), 100U/ml penicillin and 100μg/ml streptomycin and cultured on coverslips coated with poly-L-lysine (100μg/ml) in a humidified CO2 incubator. Nerve growth factor (NGF) was not added to the culture to prevent unexpected effects.

For imaging DRG neurons isolated from mice with CFA inflammatory pain and SNI neuropathic pain, only lumbar DRG (L3-L5) from the contralateral and ipsilateral sides were isolated. Dissociated DRG neurons were plated in droplet on the coverslips coated with 100μg/ml poly-l-lysine. Neurons were allowed to adhere for 1h at 37°C before adding more DMEM medium containing 2%FBS, 100U/ml penicillin and 100μg/ml streptomycin. DRG neurons were then immediately used for calcium imaging within 5h after isolation.

Ca^{2+} imaging

DRG neurons on coverslips were loaded with cell permeable Fura-2AM (ThermoFisher, 3.3μM) at 37°C for 20min. Coverslips were then mounted in a perfusion chamber. The chamber was transferred to the imaging stage of a Nikon inverted microscope and connected to an automated perfusion system (Warner Instruments). Coverslip in the chamber was continuously perfused with Hanks' balanced salt solution containing (in mM) 140 NaCl, 4 KCl, 10 HEPES, 1.8 $CaCl_2$, 1$MgCl_2$, 5 Glucose (pH 7.4). Fura-2AM loaded cells were exposed alternatively for 50ms (exposure time) to 340nm and 380nm LED illuminator (Cairn Research UK). Emission was collected every two seconds at 510nm using a sCMOS camera (Photometrics). Image acquisition and solution perfusion were controlled by the MetaFluor software (Molecular

Devices). Ratio of emission signals of 340nm and 380nm were calculated as an index of $[Ca^{2+}]_i$. Different drugs were delivered to the perfusion chamber through manifold. Neurons were identified by their responses to 50mM KCl. A 10% increase in fluorescence ratio over baseline was considered as a response. We quantified the percentage of responding neurons by calculating the ratio of the number of responding neurons evoked by low dose AITC to the total number of TRPA1$^+$ DRG neurons evoked by saturating dose AITC.

Membrane protein detection and western blotting

Membrane proteins were labelled using biotinylation assay as described previously[50]. Briefly, HEK293 cells expressing TRPA1 and other cDNA constructs were live labelled with 2mM EZ-link Sulfo-NHS-LC-Biotin (ThermoFisher) on ice for one hour after stimulation. Cells were then washed with cold PBS containing 100mM glycine followed by solubilization using a lysis buffer consisting of 20mM HEPES (pH 7.4), 150mM NaCl, 1mM EDTA, 1mM EGTA, 1% NP-40 plus protease inhibitor cocktails (Merck). Cell lysate was incubated at 4°C for 30min on a mixer with constant rotation followed by centrifuge at 12,000rpm for 10min at 4°C. Supernatant was then incubated with Streptavidin agarose (Pierce) at 4°C overnight. Proteins were then thoroughly washed with lysis buffer succeeded by boiling in laemmli buffer. Proteins were next separated in 7.5% SDS-PAGE gel and transferred to a PVDF blot (ThermoFisher). Membrane TRPA1 was finally detected by anti-V5 (ThermoFisher).

We also isolated membrane protein using Mem-PER Plus Membrane Protein Extraction Kit (ThermoFisher) from DRG and sciatic nerves in accordance with instructions with mild modifications. Briefly, lumbar DRG (L3-L5) was washed with 200µl wash buffer after isolation. They were then added to 200µl permeabilization buffer and homogenized using a motor-driven homogenizer followed by incubation at 4°C for 10min. Cell suspension was centrifuged at 16,000rpm for 15min at 4°C. Cell pellet was next resuspended in the solubilization buffer containing protease inhibitors and homogenized with a homogenizer. Cell solution was then incubated at 4°C for 30min with constant mixing prior to centrifuge at 16,000rpm for 15min at 4°C. Supernatant was then used for Western blotting as described above. Membrane TRPA1 was detected using anti-TRPA1 antibody (Alomone labs).

Pull down assay, coimmunoprecipitation and proteomics

Nickel bread pull down assay was used to detect interaction between TRPA1 and IQGAP1 in HEK293 cells and was performed as described previously with modificatons[52]. Briefly, HEK293 cells transfected with TRPA1-V5-Histidine tag and IQGAP1 or other IQGAP1 deletion constructs (ΔCaM-IQGAP1, ΔMK24-IQGAP1) were solubilized in lysis buffer containing 20mM Tris-HCl (pH 7.4), 300mM NaCl, 1% NP-40, 0.4mM EDTA, 20mM Imidazole, 10% Glycerol plus protease inhibitor cocktails. Cell lysate was then incubated at 4°C for 30min before centrifuge at 12,000rpm for 10min at 4°C. Supernatant of cell lysate was then incubated with 30μl Ni-NTA beads (Qiagen) at 4°C overnight. Nickel beads were then thoroughly washed in lysis buffer before subjected to boiling in sample buffer. Protein supernatant was then separated in 7.5%SDS-PAGE gel followed by blot transfer and protein detection using anti-IQGAP1(Santa Cruz) and anti-V5 (ThermoFisher).

Nickel beads purified proteins from HEK293 cells expressing TRPA1-V5-6×histidine were also used for identification of unknown proteins copurified with TRPA1 (Fig. 2A). Briefly, after separation in 7.5% SDS-PAGE gel, purified proteins were fixed and stained with silver staining kit (Sigma) in accordance with manufacturer's instructions. Protein bands of interest were excised and processed for LC-MS/MS analysis in the proteomic centre at the University of Aberdeen.

GST pull down assay was employed to delineate the binding regions between TRPA1 and IQGAP1 and was conducted as described previously[52, 53]. Briefly, the cytoplasmic tails of TRPA1 coupled to GST tag were purified from HEK293 cells expressing the fusion proteins using GST-agarose (Merck). Purified GST-coupled fusion proteins were then incubated with HEK293 cell lysate containing IQGAP1 or N-IQGAP1 or Flag-C-IQGAP1 at 4°C overnight with constant mixing. Bound proteins were then dissociated from beads by boiling in Laemmli buffer followed by protein separation and detection using anti-IQGAP1 and/or -Flag (Merck).

Co-immunoprecipitation was used to detect TRPA1-IQGAP1 interaction in DRG neurons and binding of HA-CaM to IQGAP1 in HEK293 cells. It was performed as described previously[53]. In Brief, DRG neurons were solubilized in lysis buffer. Cell lysate was then isolated and incubated with monoclonal anti-IQGAP1 or anti-HA (Covance) and Protein A/G PLUS-agarose (Santa Cruz) at 4°C overnight. Protein-bound beads were thoroughly washed before

boiling in Laemmli buffer. Dissociated proteins were next separated in SDS-PAGE gel followed by blot transfer and protein detection using anti-TRPA1 (Alomone) or anti-IQGAP1.

Immunohistochemistry and immunocytochemistry

Mice were transcardially perfused with PBS and 4% paraformaldehyde (PFA). Lumbar DRG (L4-L5), sciatic nerve and skin were then isolated and post-fixed in 4% PFA at 4°C overnight with constant rotation followed by cryopreservation in 30% sucrose. Tissues were embedded in OCT medium (Tissue-Tek) and sectioned in a cryostat at 12μm thickness. DRG sections were placed onto poly-lysine-coated slides for immunohistochemistry. DRG tissue sections were first blocked in 5% Donkey serum plus 0.03% Triton X-100 at room temperature for 30min. They were then incubated with primary antibodies (mouse anti-IQGAP1(Santa Cruz), rabbit anti-TRPA1 (Alomone), rabbit anti-CGRP (Merck) and rabbit anti-TRPV1 (Santa Cruz)) at 4°C overnight. After wash in PBS, DRG sections were next incubated with fluorescence-conjugated secondary antibodies (Alexa Fluro 594 conjugated donkey anti-mouse, Alexa Fluor 488 conjugated chicken anti-rabbit and FITC-conjugated Isolectin B4 (Merck)) at Rt for 2h. After thorough wash, DRG slides were sealed by coverslips with Mowoil solution.

Immunocytochemistry and live labelling

Cultured DRG neurons were fixed in 4%PFA at RT for 20min followed by blocking in 0.1% Gelatin (Merck) for 1h. To label membrane TRPA1, DRG neurons were incubated with rabbit anti-TRPA1 antibody binding to an extracellular epitope of TRPA1 at 4°C overnight under nonpermeabilized condition. DRG neurons were then permeabilized in 0.1%Triton X-100 for 15min at RT followed by incubation with mouse anti-IQGAP1 at 4°C overnight. After wash, cells were next incubated with Alexa Fluor 488 conjugated chicken anti-rabbit and Alexa Fluor 594 conjugated donkey anti-mouse secondary antibodies at RT for 1h. DRG neurons were finally mounted onto slides with Mowoil solution for examination with Confocal microscopy.

Membrane TRPA1 in DRG neurons was also live labelled as described[37]. Briefly, DRG neurons were incubated with anti-TRPA1 (1:25 dilution) directed against an extracellular epitope of TRPA1 at 37°C for 10min. After wash in serum free DMEM medium, DRG neurons

were incubated with Alexa Fluor 488 conjugated anti-rabbit secondary antibody at RT for 20min. To label cytoplasmic IQGAP1, neurons were further fixed in 4%PFA and permeabilized in 0.1% Triton X-100 followed by incubation with anti-IQGAP1 primary antibody and corresponding secondary antibody as described above.

Quantitative real-time RT-PCR (qPCR)

L3-L5 lumbar DRG was rapidly isolated 7 days after CFA injection (i.pl) or SNI surgery. Total RNA was extracted from lumbar DRG using TRIzol reagent (Invitrogen) and reverse transcribed to cDNA using SuperScript^{TM} II reverse transcriptase (Invitrogen) in accordance with the manufacturer's instructions. qPCR reactions were prepared by mixing cDNA with SYBR green master mix reagents (Applied Biosystems) and primer pairs (300nM each) in a 384-well plate with every reaction in triplicate. Primers used for IQGAP1 amplification: forward: 5'- AGAAAGCTGTGGCAATTCAA-3'; reverse: 5'-TTGTTGGTCAACGTGAGAGA-3'. TRPA1: forward: 5'-AGTGGCAATGTGGAGCAATA -3'; reverse: 5'-AACACTCCGGTCGATCTCA -3'. GAPDH: forward: 5'-CCAAGGTCATCCATGACAAC-3'; reverse: 5'-GGATGCAGGGATGATGTTCT-3'. cDNA was amplified in LightCycler 480 (Roche). All gene expression was normalised against GAPDH and analysed using ΔΔCt method.

Electrophysiology

Whole-cell electrophysiology recordings were performed at RT as described previously[51]. In brief, small-diameter DRG neurons were recorded with patch pipettes fabricated from thin-walled glass capillary using a pipette puller (Sutter Instrument) with a resistance between 2.5~4.0MΩ. Pipette solution contains (in mM): 140 KCl, 2.0 $MgCl_2$, 5 EGTA and 10 HEPES, pH7.4 with KOH. Neurons were perfused with extracellular solution consisting of (in mM): 140 NaCl, 4KCl, 10 HEPES, 1 MgCl2, 5 EGTA and 5 Glucose, pH7.4 with NaOH. Series resistance was 80% compensated. Signals were analog filtered at 1KHz using a low-pass Bessel filter of the amplifier and digitized using Digidata 1440A (Molecular Devices).

Statistics

All data are mean ± SEM. Significance between groups was determined using Student's *t* test or one or two-way ANOVA followed by Bonferroni post-hoc test. $P<0.05$ was considered to be significant.

Supplementary Fig. 1

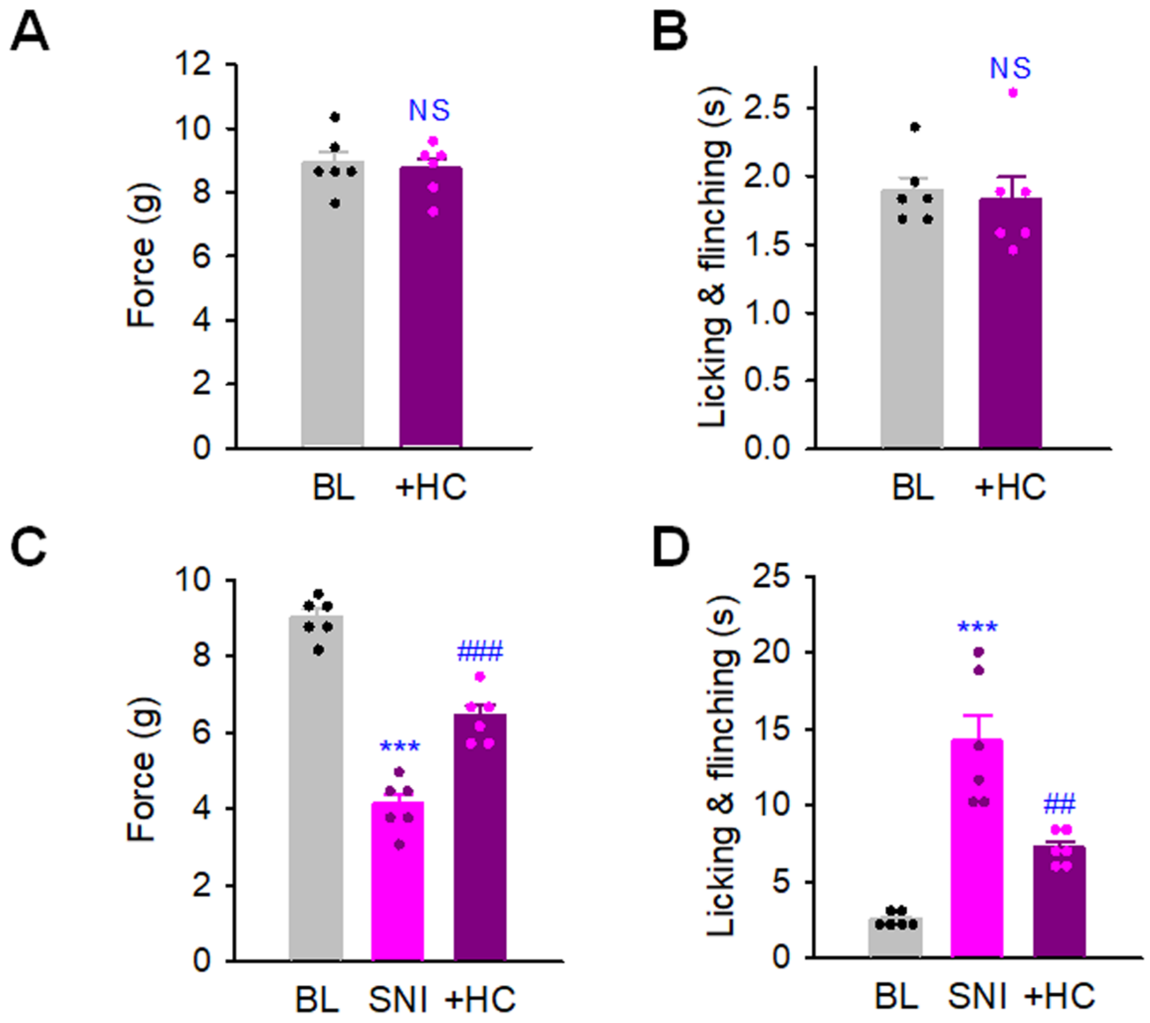

Supplementary Fig. 1. Blockade of TRPA1 reduced chronic mechanical and cold pain in SNI model. Related to Fig. 1. (A, B) Threshold mechanical force to paw withdrawal in Von Frey test **(A)** and time spend on licking and flinching in response to acetone evaporation **(B)** under baseline (BL) and after injection (i.pl) with HC-030031 (1mM, 10μl). n=6 per group. NS, not significant. **(C, D)** Paw withdrawal force **(C)** and duration of licking and flinching in response to acetone **(D)** in mice under baseline (BL) and 14 days after SNI surgery or SNI mice pre-injected (i.pl) with HC-030031. n=6 per group. ***P<0.001 compared to BL; ##P<0.01 compared to SNI group.

Supplementary Fig. 2

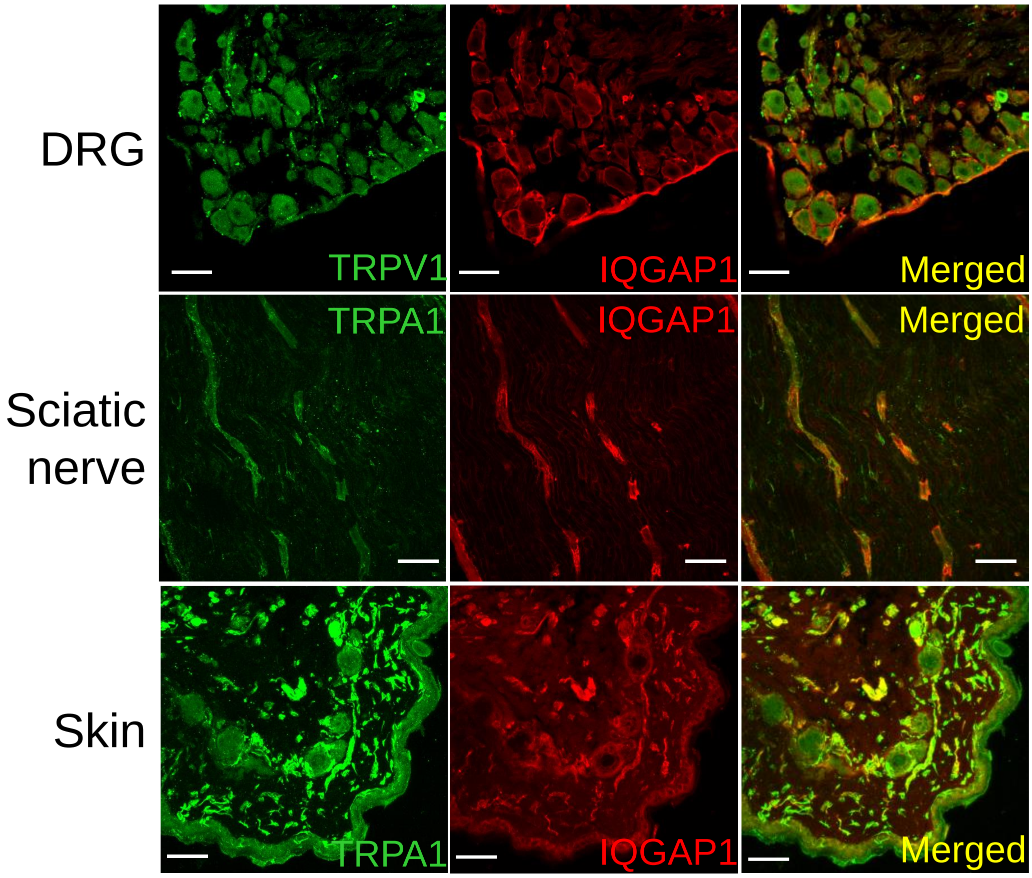

Supplementary Fig. 2. Related to Fig. 2. Co-expression of IQGAP1 with TRPV1 in lumbar DRG, sciatic nerve and skin in mice. Scale bars, 50µm.

Supplementary Fig. 3

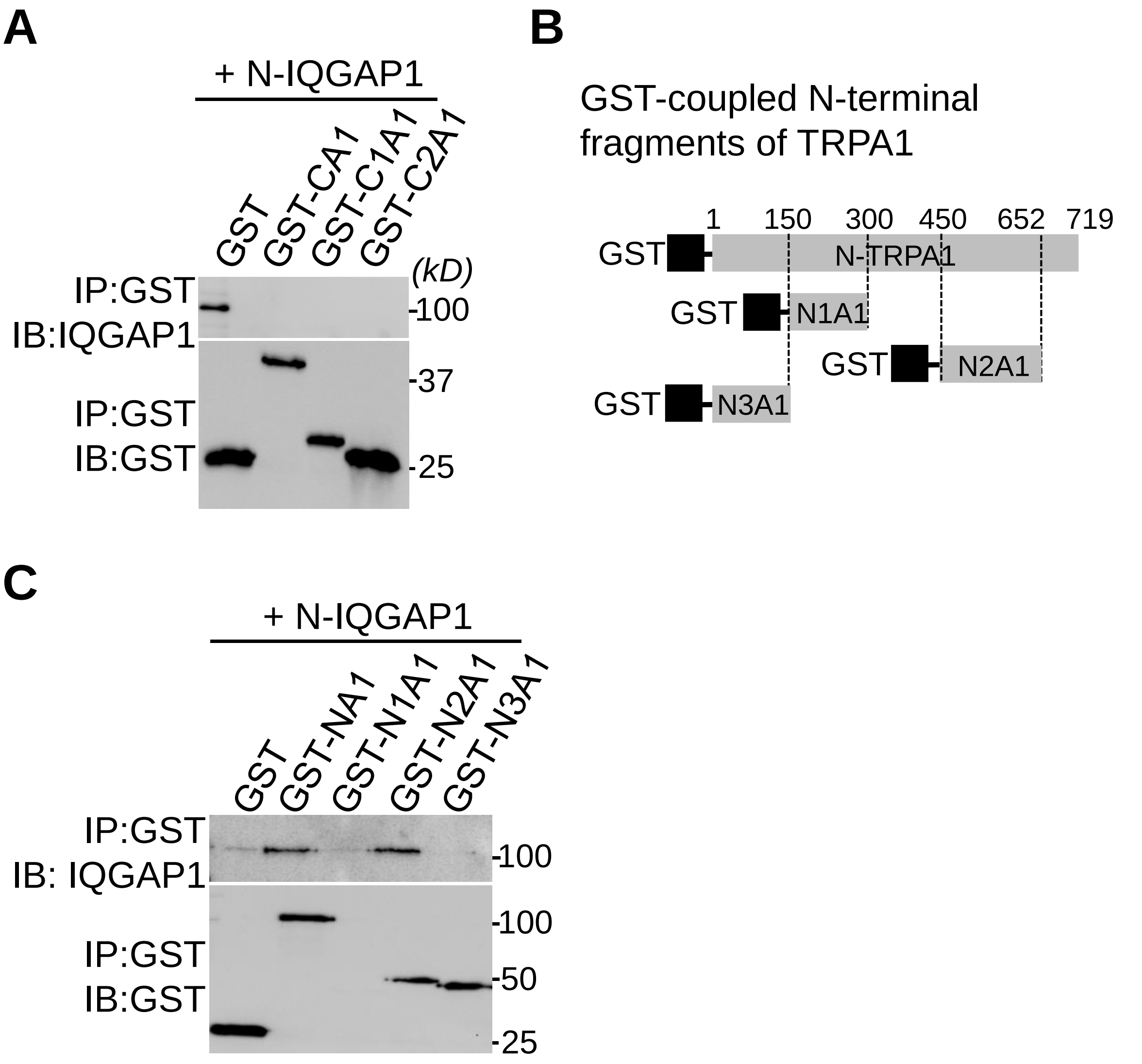

Supplementary Fig. 3. Molecular delineation of mutual interactions between the N- and C-termini of TRPA1 and IQGAP1. Related to Fig. 3. (A) The C-terminal fragments of TRPA1 (showing in bottom blot) does not bind to N-IQGAP1 in GST pull down assay (top blot). **(B)** Schematic diagram shows different fragments derived from the N-terminus of TRPA1 coupled to GST tag. **(C)** GST pull down assay shows the binding of N-IQGAP1 to the N-terminal fragments of TRPA1 depicted in B. All the blots were repeated at least three times.

Supplementary Fig. 4

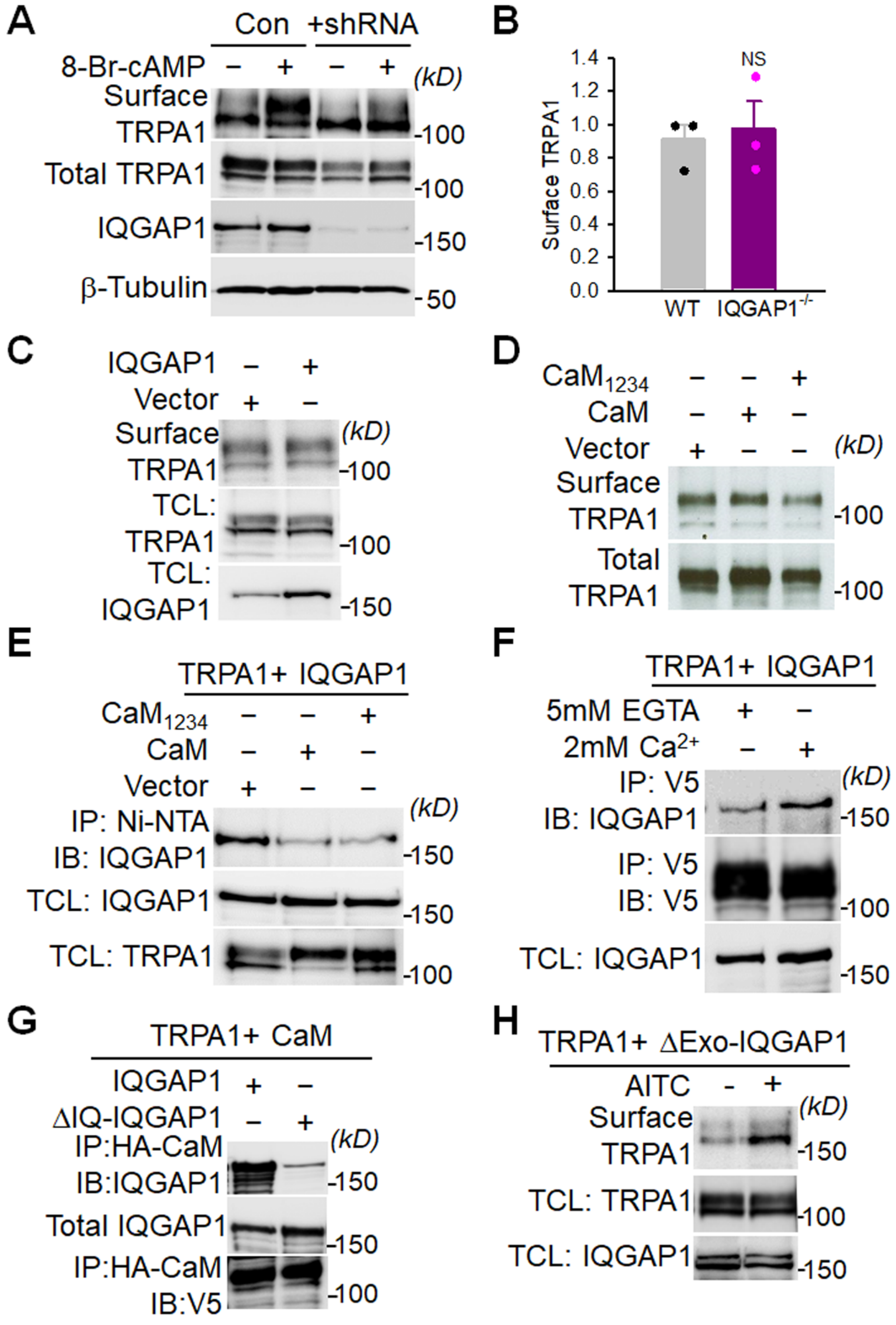

Supplementary Fig. 4. Regulation of TRPA1 trafficking and IQGAP1 binding by Ca^{2+}/CaM. Related to Fig. 4 and 5. (A) Biotinylation of membrane TRPA1 in HEK293 cells expressing TRPA1 or in cells expressing IQGAP1 shRNA. IQGAP1 is knocked down by shRNA (seethe third blot). Band densities relative to the first band in the top blot: 1.0, 1.34, 0.90, 1.10. Total TRPA1 and tubulin are comparable between different groups. **(B)** Summary of normalised membrane TRPA1 expression in wild-type (WT) and IQGAP1-lacking HEK293 cells expressing TRPA1 from experiments similar to those in Fig. 4C & 4D. n=3, NS, not significant. **(C)** Overexpression of IQGAP1 (showing in bottom blot) does not affect the basal membrane TRPA1 (top blot). TRPA1 expression in total cell lysate (TCL) is similar (middle blot). Band densities 1.0, 0.99. **(D)** Membrane TRPA1 is not affected by overexpression CaM or CaM_{1234} (top blot). Band densities: 1.0, 1.01, 0.88. **(E)** Nickel beads pull down assay shows that CaM and CaM_{1234} reduce the binding of TRPA1 to IQGAP1 (top blot). Band densities 1.0, 0.40, 0.46. Total IQGAP1 and TRPA1 are comparable in all samples (middle and bottom blots). **(F)** ΔIQ-IQGAP1 exhibits a robust reduction in the binding to CaM (top blot), though it does not affect binding between TRPA1 and CaM (bottom blot). Band densities 1.0, 1.54. **(G)** ΔExo-IQGAP1 does not affect increased TRPA1 trafficking caused by AITC (400μM, 4 min) (top blot). Total TRPA1 and IQGAP1 expression are similar in all the samples (middle and bottom lots). Band densities 1.0, 0.31. **(H)** TRPA1 binds to more IQGAP1 in the presence of Ca^{2+} than in Ca^{2+}-free revealed in a nickel beads pull down assay (top blot). Band densities 1.0, 2.08. All the blots were repeated at least three times.

Supplementary Fig. 5

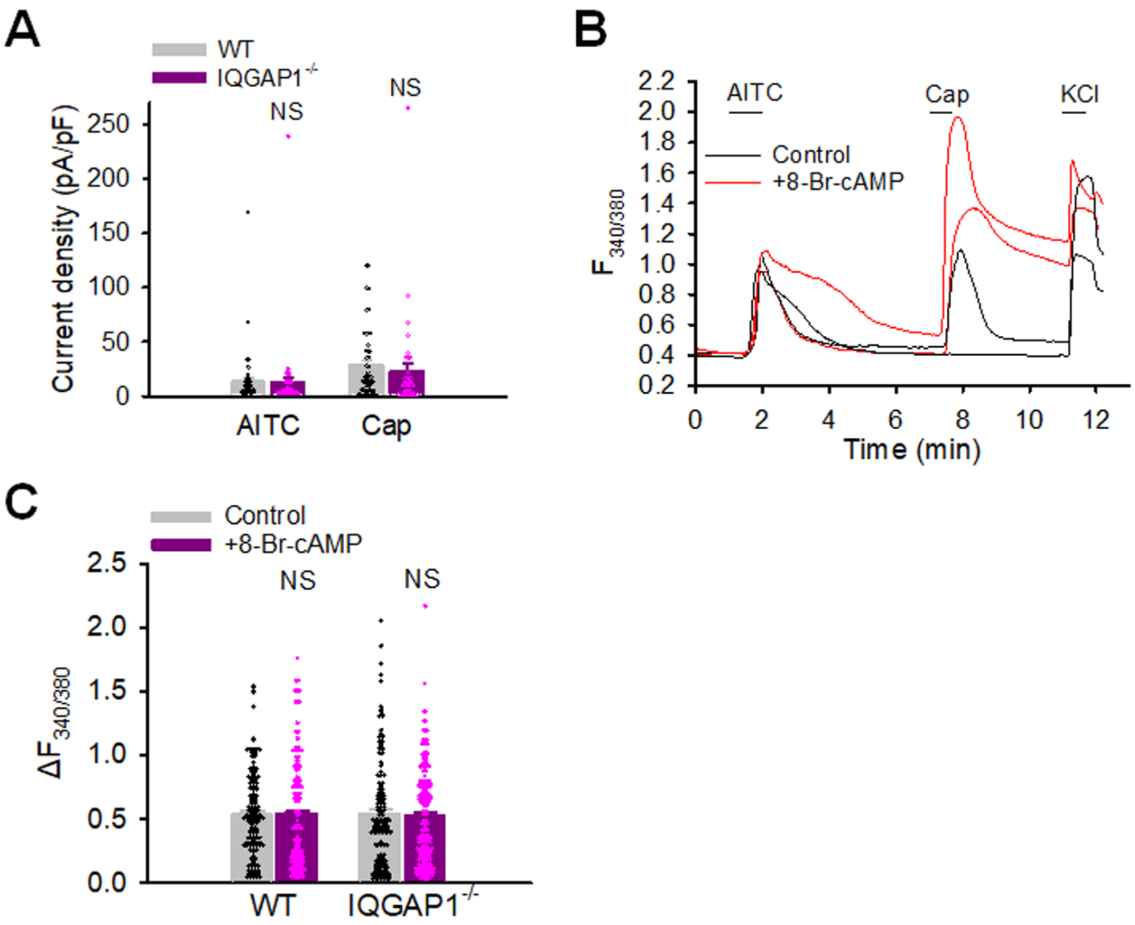

Supplementary Fig. 5. PKA does not affect the maximal peak TRPA1 responses in DRG neurons. Related to Fig. 6. (A) Summary of current density of TRPA1 and TRPV1 channels in WT and IQGAP1$^{-/-}$ DRG neurons activated by AITC (100μM, 20s) and Capsaicin (1μM, 5s). n_{cell}=38~49 per group. NS, not significant. **(B)** Example traces of DRG neurons responding to AITC (100μM), Capsaicin (Cap, 1μM) and KCl (50mM) in control (in black) and treated with (in red) 8-Br-cAMP (50μM, 10min). **(C)** Summary of peak Ca^{2+} responses in WT and IQGAP1-lacking DRG neurons evoked by AITC from experiments similar to those in **B**. n=3, n_{cell}=115~152 per group. NS, not significant.

Supplementary Fig. 6

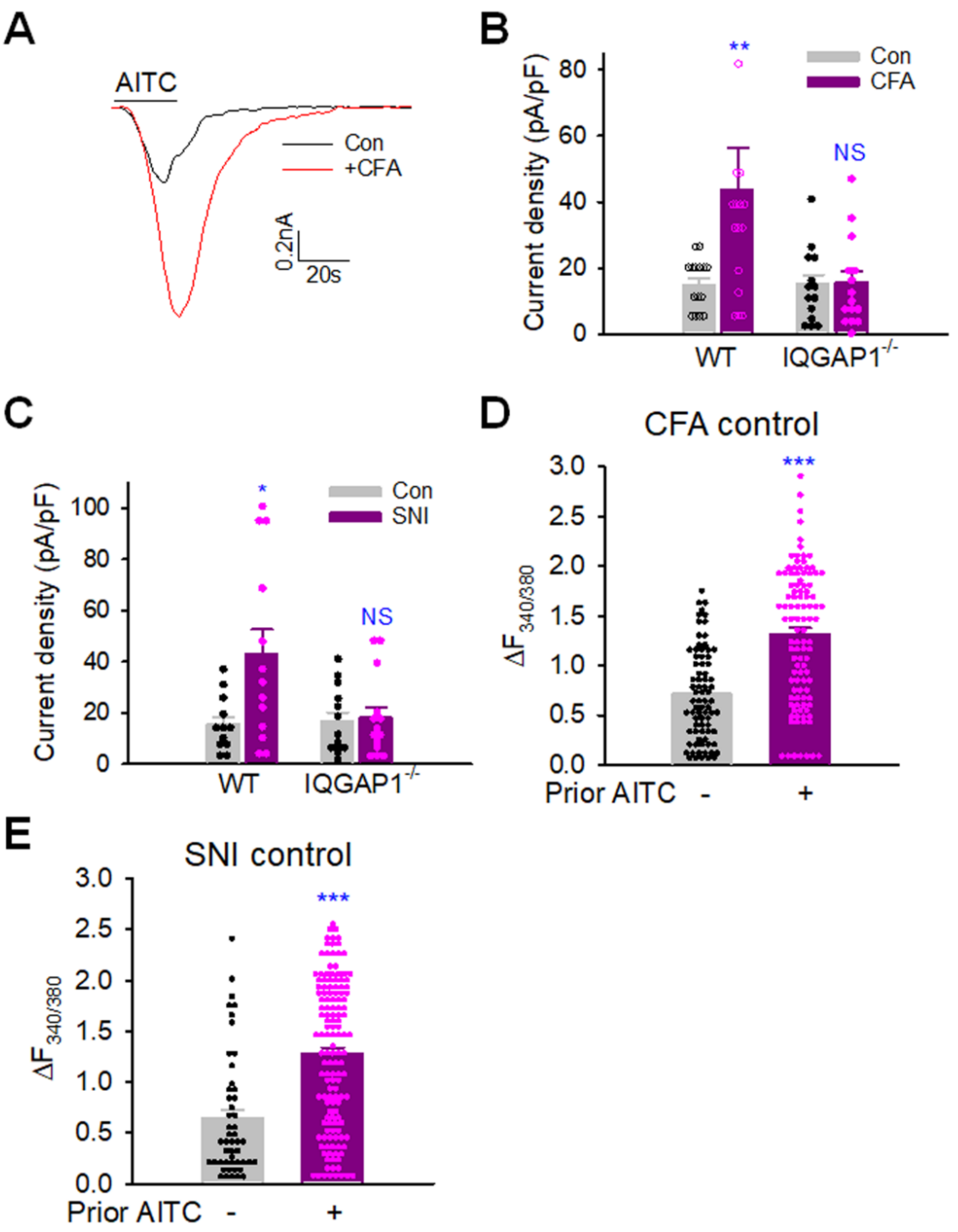

Supplementary Fig. 6. Related to Fig. 7. (A) Example TRPA1 currents evoked by AITC (100µM) in the contralateral (Con) and ipsilateral DRG acutely dissociated from mice 7 days after injection with CFA. **(B, C)** Summary of peak TRPA1 current density in DRG from CFA (**B**) and SNI (**C**) mice from experiments similar to those in A. Note that a current density of 222.8pA/pF in WT SNI group was not depicted in the graph due to beyond the scale of the graph. n_{cell}=12~16 per group. **P*<0.05; ***P*<0.01; NS, not significant. **(D, E)** Summary of peak Ca^{2+} responses evoked by AITC (100µM) in DRG neurons with or without prior activation by AITC (5µM) in the contralateral DRG neurons (control) from CFA (**E**) and SNI (**F**) mice from experiments similar to those in Fig. 7A, 7E (CFA model), 7G & 7K (SNI model). n_{cell}=49~139 per group. ****P*<0.001.

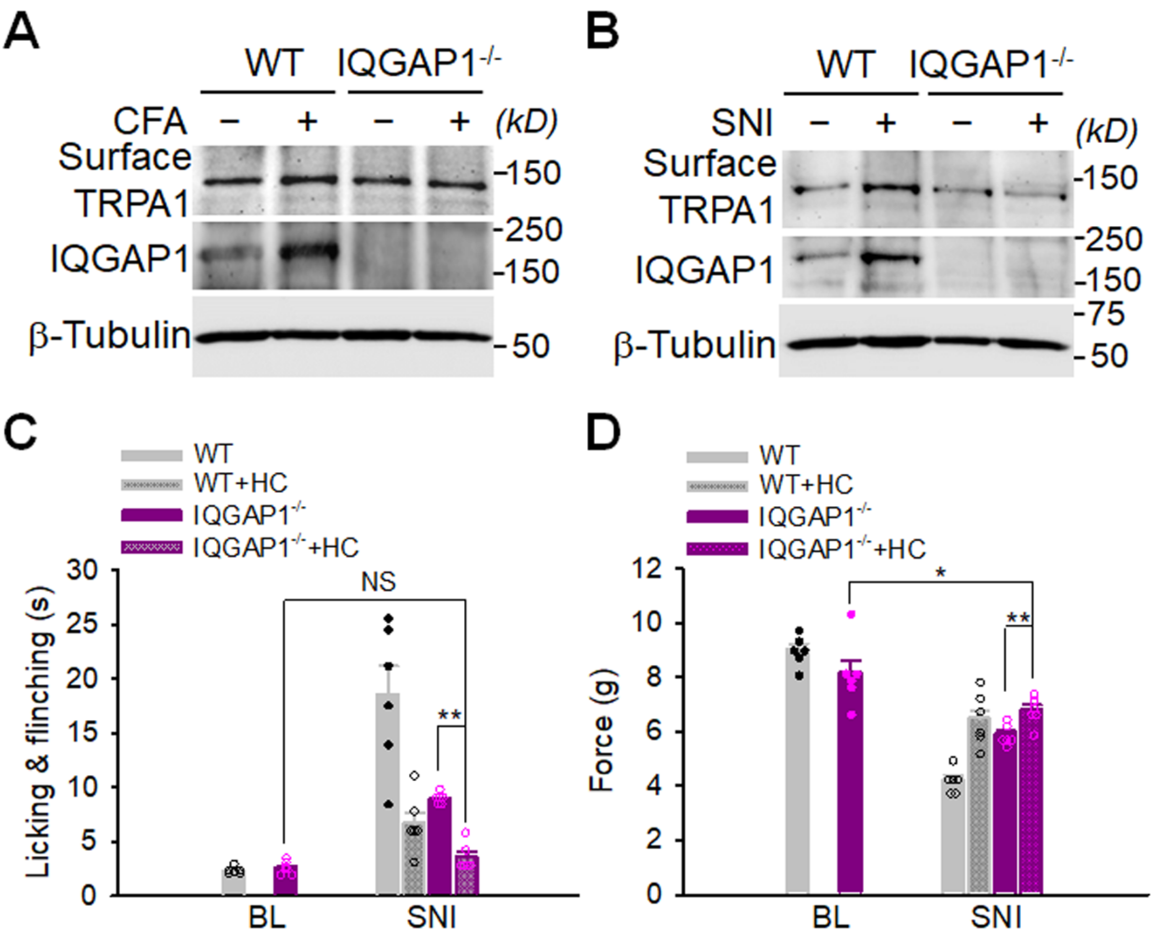

Supplementary Fig. 7. TRPA1 trafficking is critical to chronic cold and mechanical pain. Related to Fig. 8. (A, B) Surface TRPA1 in sciatic nerve isolated from the contralateral and ipsilateral sides of WT and IQGAP1$^{-/-}$ mice injected with CFA **(A)** or with SNI surgery. Relative band densities in the top blot in **A**: 1.0, 1.13, 1.0, 1.01; **B**: 1.0, 1.69, 1.06, 0.91. **(B)**. IQGAP1 is increased in sciatic nerve in CFA and SNI mice. Total tubulin is comparable in all conditions. **(C, D)** Duration of licking and flinching in acetone evaporation assay **(C)** and paw withdrawal force **(D)** under baseline (BL) and 7 days after SNI surgery in WT and IQGAP1$^{-/-}$ mice. Some mice were also injected (i.pl) with HC-030031 (1mM, 10μl). n=6 per group. *P<0.05; **P<0.01; NS, not significant.

Supplementary table

A list of protein candidates potentially binding to TRPA1 revealed by LC-MS/MS analysis

LC-MS/MS analysis of TRPA1 binding proteins - related to Figure 2A

Accession	Description	Score	Coverage	# Proteins	Unique Peptide	# Peptides	# PSMs	Area	# AAs	MW [kDa]	calc. pI
P46940	Ras GTPase-activating-like protein IQGAP1 OS=Homo sapiens GN=IQGAP1 PE=1 SV=1 - [IQGA1_HUMAN]	4212.73	57.63	1	76	76	129	5.650E8	1657	189.1	6.48
Q96CP6	GRAM domain-containing protein 1A OS=Homo sapiens GN=GRAMD1A PE=1 SV=2 - [GRM1A_HUMAN]	1124.51	46.69	1	21	24	33	3.570E8	724	80.6	6.74
O75762	Transient receptor potential cation channel subfamily A member 1 OS=Homo sapiens GN=TRPA1 PE=1 SV=3 - [TRPA1_HUMAN]	450.62	9.38	1	7	7	19	3.484E8	1119	127.4	7.12
O76024	Wolframin OS=Homo sapiens GN=WFS1 PE=1 SV=2 - [WFS1_HUMAN]	1090.33	28.99	1	17	17	31	3.308E8	890	100.2	8.05
P04264	Keratin, type II cytoskeletal 1 OS=Homo sapiens GN=KRT1 PE=1 SV=6 - [K2C1_HUMAN]	973.26	29.04	1	14	16	24	2.692E8	644	66.0	8.12
Q96T76	MMS19 nucleotide excision repair protein homolog OS=Homo sapiens GN=MMS19 PE=1 SV=2 - [MMS19_HUMAN]	1467.85	38.74	1	27	27	39	2.132E8	1030	113.2	6.35
P35908	Keratin, type II cytoskeletal 2 epidermal OS=Homo sapiens GN=KRT2 PE=1 SV=2 - [K22E_HUMAN]	335.98	14.40	1	3	7	7	1.766E8	639	65.4	8.00
Q3KR37	GRAM domain-containing protein 1B OS=Homo sapiens GN=GRAMD1B PE=1 SV=1 - [GRM1B_HUMAN]	165.58	11.79	1	4	7	7	1.263E8	738	85.3	6.21
Q8TB52	F-box only protein 30 OS=Homo sapiens GN=FBXO30 PE=1 SV=3 - [FBX30_HUMAN]	749.66	38.26	1	19	19	30	1.233E8	745	82.3	5.40
P35527	Keratin, type I cytoskeletal 9 OS=Homo sapiens GN=KRT9 PE=1 SV=3 - [K1C9_HUMAN]	665.32	35.96	1	16	16	20	1.093E8	623	62.0	5.24
Q08J23	tRNA (cytosine(34)-C(5))-methyltransferase OS=Homo sapiens GN=NSUN2 PE=1 SV=2 - [NSUN2_HUMAN]	674.48	39.63	1	20	20	28	9.039E7	767	86.4	6.77
P13645	Keratin, type I cytoskeletal 10 OS=Homo sapiens GN=KRT10 PE=1 SV=6 - [K1C10_HUMAN]	604.28	29.28	1	12	12	14	7.576E7	584	58.8	5.21
P27824	Calnexin OS=Homo sapiens GN=CANX PE=1 SV=2 - [CALX_HUMAN]	532.94	22.97	1	11	11	14	7.440E7	592	67.5	4.60
O00159	Unconventional myosin-Ic OS=Homo sapiens GN=MYO1C PE=1 SV=4 - [MYO1C_HUMAN]	1065.65	28.88	1	23	24	29	6.560E7	1063	121.6	9.41
O00418	Eukaryotic elongation factor 2 kinase OS=Homo sapiens GN=EEF2K PE=1 SV=2 - [EF2K_HUMAN]	568.21	25.38	1	14	14	25	6.223E7	725	82.1	5.33
Q92562	Polyphosphoinositide phosphatase OS=Homo sapiens GN=FIG4 PE=1 SV=1 - [FIG4_HUMAN]	461.35	28.67	1	18	18	20	6.206E7	907	103.6	6.92
O43795	Unconventional myosin-Ib OS=Homo sapiens GN=MYO1B PE=1 SV=3 - [MYO1B_HUMAN]	711.65	24.03	1	20	21	22	3.317E7	1136	131.9	9.38
P13647	Keratin, type II cytoskeletal 5 OS=Homo sapiens GN=KRT5 PE=1 SV=3 - [K2C5_HUMAN]	312.50	21.19	1	8	10	10	2.904E7	590	62.3	7.74
Q6P5Z2	Serine/threonine-protein kinase N3 OS=Homo sapiens GN=PKN3 PE=1 SV=1 - [PKN3_HUMAN]	396.08	24.30	1	15	15	15	2.892E7	889	99.4	8.46
O94832	Unconventional myosin-Id OS=Homo sapiens GN=MYO1D PE=1 SV=2 - [MYO1D_HUMAN]	617.61	24.06	1	19	20	24	2.627E7	1006	116.1	9.39
Q9NRF2	SH2B adapter protein 1 OS=Homo sapiens GN=SH2B1 PE=1 SV=3 - [SH2B1_HUMAN]	397.61	27.91	1	11	11	11	2.621E7	756	79.3	5.38
O00469	Procollagen-lysine,2-oxoglutarate 5-dioxygenase 2 OS=Homo sapiens GN=PLOD2 PE=1 SV=2 - [PLOD2_HUMAN]	225.55	20.35	1	10	10	11	2.111E7	737	84.6	6.71
Q9UM54	Unconventional myosin-VI OS=Homo sapiens GN=MYO6 PE=1 SV=4 - [MYO6_HUMAN]	467.95	15.84	1	14	14	17	2.056E7	1294	149.6	8.53
Q9NRZ9	Lymphoid-specific helicase OS=Homo sapiens GN=HELLS PE=1 SV=1 - [HELLS_HUMAN]	289.98	11.81	1	9	9	11	2.007E7	838	97.0	7.93
P02533	Keratin, type I cytoskeletal 14 OS=Homo sapiens GN=KRT14 PE=1 SV=4 - [K1C14_HUMAN]	173.39	14.83	1	6	6	7	1.841E7	472	51.5	5.16
Q96DU7	Inositol-trisphosphate 3-kinase C OS=Homo sapiens GN=ITPKC PE=1 SV=1 - [IP3KC_HUMAN]	184.91	13.32	1	6	6	7	1.795E7	683	75.2	5.14
Q12965	Unconventional myosin-Ie OS=Homo sapiens GN=MYO1E PE=1 SV=2 - [MYO1E_HUMAN]	395.57	12.45	1	10	11	12	1.639E7	1108	127.0	8.92
P53618	Coatomer subunit beta OS=Homo sapiens GN=COPB1 PE=1 SV=3 - [COPB_HUMAN]	301.38	20.36	1	12	12	12	1.526E7	953	107.1	6.05
Q8IY17	Neuropathy target esterase OS=Homo sapiens GN=PNPLA6 PE=1 SV=2 - [PLPL6_HUMAN]	201.13	7.10	1	7	7	7	1.491E7	1366	149.9	7.81
Q9UHB6	LIM domain and actin-binding protein 1 OS=Homo sapiens GN=LIMA1 PE=1 SV=1 - [LIMA1_HUMAN]	99.77	5.14	1	3	3	3	1.467E7	759	85.2	6.84
P13639	Elongation factor 2 OS=Homo sapiens GN=EEF2 PE=1 SV=4 - [EF2_HUMAN]	294.93	12.12	1	8	8	8	1.446E7	858	95.3	6.83
O43592	Exportin-T OS=Homo sapiens GN=XPOT PE=1 SV=2 - [XPOT_HUMAN]	442.74	14.97	1	11	11	13	1.425E7	962	109.9	5.39
P29590	Protein PML OS=Homo sapiens GN=PML PE=1 SV=3 - [PML_HUMAN]	184.08	5.90	1	4	4	4	1.337E7	882	97.5	6.21
Q12979	Active breakpoint cluster region-related protein OS=Homo sapiens GN=ABR PE=2 SV=2 - [ABR_HUMAN]	234.47	12.11	1	7	7	9	1.234E7	859	97.5	6.55
O14795	Protein unc-13 homolog B OS=Homo sapiens GN=UNC13B PE=1 SV=2 - [UN13B_HUMAN]	272.08	9.18	1	10	10	11	1.183E7	1591	180.6	5.99
Q9H0W5	Coiled-coil domain-containing protein 8 OS=Homo sapiens GN=CCDC8 PE=1 SV=2 - [CCDC8_HUMAN]	222.35	16.36	1	4	4	6	1.177E7	538	59.3	8.63
P56192	Methionine--tRNA ligase, cytoplasmic OS=Homo sapiens GN=MARS PE=1 SV=2 - [SYMC_HUMAN]	263.95	11.67	1	8	8	8	1.115E7	900	101.1	6.16
Q01813	ATP-dependent 6-phosphofructokinase, platelet type OS=Homo sapiens GN=PFKP PE=1 SV=2 - [PFKAP_HUMAN]	273.43	17.47	1	10	10	11	1.097E7	784	85.5	7.55
Q14244	Ensconsin OS=Homo sapiens GN=MAP7 PE=1 SV=1 - [MAP7_HUMAN]	85.56	6.28	1	4	4	4	1.053E7	749	84.0	9.61
P42704	Leucine-rich PPR motif-containing protein, mitochondrial OS=Homo sapiens GN=LRPPRC PE=1 SV=3 - [LPPRC_HUMAN]	265.03	10.55	1	10	10	10	1.014E7	1394	157.8	6.13
Q27J81	Inverted formin-2 OS=Homo sapiens GN=INF2 PE=1 SV=2 - [INF2_HUMAN]	46.71	3.04	1	3	3	3	9.534E6	1249	135.5	5.38
Q9H3U1	Protein unc-45 homolog A OS=Homo sapiens GN=UNC45A PE=1 SV=1 - [UN45A_HUMAN]	310.16	14.19	1	11	11	11	9.148E6	944	103.0	6.07
Q3B820	Protein FAM161A OS=Homo sapiens GN=FAM161A PE=1 SV=2 - [F161A_HUMAN]	189.18	13.03	1	5	5	5	8.613E6	660	76.7	8.03
A1L390	Pleckstrin homology domain-containing family G member 3 OS=Homo sapiens GN=PLEKHG3 PE=1 SV=1 - [PKHG3_HUMAN]	60.14	4.10	1	3	3	3	8.213E6	1219	134.3	6.55
O95071	E3 ubiquitin-protein ligase UBR5 OS=Homo sapiens GN=UBR5 PE=1 SV=2 - [UBR5_HUMAN]	237.30	4.68	1	9	9	9	8.106E6	2799	309.2	5.85
Q14573	Inositol 1,4,5-trisphosphate receptor type 3 OS=Homo sapiens GN=ITPR3 PE=1 SV=2 - [ITPR3_HUMAN]	117.19	2.92	1	6	6	6	7.950E6	2671	303.9	6.48
Q5VUJ6	Leucine-rich repeat and calponin homology domain-containing protein 2 OS=Homo sapiens GN=LRCH2 PE=2 SV=2 - [LRCH2_HU	127.24	8.37	1	4	4	4	7.480E6	765	84.5	6.55
Q9ULJ3	Zinc finger and BTB domain-containing protein 21 OS=Homo sapiens GN=ZBTB21 PE=1 SV=2 - [ZBT21_HUMAN]	106.07	4.88	1	4	4	5	6.659E6	1066	118.8	8.29
O43896	Kinesin-like protein KIF1C OS=Homo sapiens GN=KIF1C PE=1 SV=3 - [KIF1C_HUMAN]	138.09	3.72	1	4	4	4	6.485E6	1103	122.9	6.90
Q9HCL2	Glycerol-3-phosphate acyltransferase 1, mitochondrial OS=Homo sapiens GN=GPAM PE=1 SV=3 - [GPAT1_HUMAN]	61.12	6.16	1	4	4	4	6.238E6	828	93.7	7.74
O75147	Obscurin-like protein 1 OS=Homo sapiens GN=OBSL1 PE=1 SV=4 - [OBSL1_HUMAN]	69.24	2.58	1	3	3	3	6.221E6	1896	206.8	5.63
Q9UBV2	Protein sel-1 homolog 1 OS=Homo sapiens GN=SEL1L PE=1 SV=3 - [SE1L1_HUMAN]	61.53	5.42	1	2	2	2	5.910E6	794	88.7	5.39
P46013	Antigen KI-67 OS=Homo sapiens GN=MKI67 PE=1 SV=2 - [KI67_HUMAN]	85.95	1.41	1	3	3	3	5.876E6	3256	358.5	9.45
Q9UHI6	Probable ATP-dependent RNA helicase DDX20 OS=Homo sapiens GN=DDX20 PE=1 SV=2 - [DDX20_HUMAN]	30.92	3.64	1	2	2	2	5.585E6	824	92.2	6.95
P43246	DNA mismatch repair protein Msh2 OS=Homo sapiens GN=MSH2 PE=1 SV=1 - [MSH2_HUMAN]	142.44	4.28	1	4	4	4	5.579E6	934	104.7	5.77
O14967	Calmegin OS=Homo sapiens GN=CLGN PE=1 SV=1 - [CLGN_HUMAN]	70.58	9.34	1	4	4	4	5.569E6	610	70.0	4.69
P55072	Transitional endoplasmic reticulum ATPase OS=Homo sapiens GN=VCP PE=1 SV=4 - [TERA_HUMAN]	109.28	12.28	1	6	6	7	5.537E6	806	89.3	5.26
P19338	Nucleolin OS=Homo sapiens GN=NCL PE=1 SV=3 - [NUCL_HUMAN]	101.33	7.75	1	5	5	5	5.468E6	710	76.6	4.70
P55060	Exportin-2 OS=Homo sapiens GN=CSE1L PE=1 SV=3 - [XPO2_HUMAN]	130.15	5.97	1	5	5	5	5.448E6	971	110.3	5.77
Q7Z7L1	Schlafen family member 11 OS=Homo sapiens GN=SLFN11 PE=1 SV=2 - [SLN11_HUMAN]	133.49	5.88	1	5	5	5	5.339E6	901	102.8	7.77
P33991	DNA replication licensing factor MCM4 OS=Homo sapiens GN=MCM4 PE=1 SV=5 - [MCM4_HUMAN]	60.98	6.26	1	4	4	4	5.026E6	863	96.5	6.74
Q08AM6	Protein VAC14 homolog OS=Homo sapiens GN=VAC14 PE=1 SV=1 - [VAC14_HUMAN]	70.42	6.65	1	3	3	3	4.972E6	782	87.9	6.13
Q13813	Spectrin alpha chain, non-erythrocytic 1 OS=Homo sapiens GN=SPTAN1 PE=1 SV=3 - [SPTN1_HUMAN]	146.53	4.49	1	7	7	7	4.969E6	2472	284.4	5.35
Q8NB90	Spermatogenesis-associated protein 5 OS=Homo sapiens GN=SPATA5 PE=1 SV=3 - [SPAT5_HUMAN]	73.99	7.28	1	4	4	4	4.785E6	893	97.8	5.66
Q14241	Transcription elongation factor B polypeptide 3 OS=Homo sapiens GN=TCEB3 PE=1 SV=2 - [ELOA1_HUMAN]	36.14	5.01	1	2	2	2	4.737E6	798	89.9	9.57
O00411	DNA-directed RNA polymerase, mitochondrial OS=Homo sapiens GN=POLRMT PE=1 SV=2 - [RPOM_HUMAN]	67.70	5.12	1	4	4	4	4.680E6	1230	138.5	8.98
O15164	Transcription intermediary factor 1-alpha OS=Homo sapiens GN=TRIM24 PE=1 SV=3 - [TIF1A_HUMAN]	114.48	3.81	1	3	3	3	4.675E6	1050	116.8	7.11
Q9Y2I7	1-phosphatidylinositol 3-phosphate 5-kinase OS=Homo sapiens GN=PIKFYVE PE=1 SV=3 - [FYV1_HUMAN]	51.82	1.43	1	3	3	3	4.429E6	2098	237.0	6.70
Q08AE8	Protein spire homolog 1 OS=Homo sapiens GN=SPIRE1 PE=1 SV=3 - [SPIR1_HUMAN]	53.38	3.84	1	3	3	3	3.911E6	756	85.5	8.62
Q68DC2	Ankyrin repeat and SAM domain-containing protein 6 OS=Homo sapiens GN=ANKS6 PE=1 SV=2 - [ANKS6_HUMAN]	47.78	4.59	1	2	2	2	3.683E6	871	92.2	7.39
P33993	DNA replication licensing factor MCM7 OS=Homo sapiens GN=MCM7 PE=1 SV=4 - [MCM7_HUMAN]	89.86	8.07	1	4	4	4	3.672E6	719	81.3	6.46
O14523	C2 domain-containing protein 2-like OS=Homo sapiens GN=C2CD2L PE=1 SV=3 - [C2C2L_HUMAN]	64.46	5.38	1	2	2	2	3.626E6	706	76.1	7.69
P35606	Coatomer subunit beta' OS=Homo sapiens GN=COPB2 PE=1 SV=2 - [COPB2_HUMAN]	71.64	4.08	1	3	3	3	3.407E6	906	102.4	5.27
Q9Y5Q9	General transcription factor 3C polypeptide 3 OS=Homo sapiens GN=GTF3C3 PE=1 SV=1 - [TF3C3_HUMAN]	118.40	5.87	1	4	4	4	3.313E6	886	101.2	5.07
O43823	A-kinase anchor protein 8 OS=Homo sapiens GN=AKAP8 PE=1 SV=1 - [AKAP8_HUMAN]	115.42	5.92	1	3	3	3	3.166E6	692	76.1	5.15
P27987	Inositol-trisphosphate 3-kinase B OS=Homo sapiens GN=ITPKB PE=1 SV=5 - [IP3KB_HUMAN]	57.33	2.22	1	2	2	2	3.133E6	946	102.3	8.43
P50570	Dynamin-2 OS=Homo sapiens GN=DNM2 PE=1 SV=2 - [DYN2_HUMAN]	47.45	6.55	1	4	4	4	3.051E6	870	98.0	7.44
Q9BZF2	Oxysterol-binding protein-related protein 7 OS=Homo sapiens GN=OSBPL7 PE=2 SV=1 - [OSBL7_HUMAN]	48.97	6.18	1	3	3	3	3.033E6	842	95.4	8.05
P28288	ATP-binding cassette sub-family D member 3 OS=Homo sapiens GN=ABCD3 PE=1 SV=1 - [ABCD3_HUMAN]	36.22	3.95	1	2	2	2	2.959E6	659	75.4	9.36
Q96PV6	Leukocyte receptor cluster member 8 OS=Homo sapiens GN=LENG8 PE=1 SV=2 - [LENG8_HUMAN]	44.39	3.34	1	2	2	2	2.956E6	779	86.1	9.35
Q86UT6	NLR family member X1 OS=Homo sapiens GN=NLRX1 PE=1 SV=1 - [NLRX1_HUMAN]	79.67	4.10	1	3	3	3	2.909E6	975	107.5	7.37
P50747	Biotin--protein ligase OS=Homo sapiens GN=HLCS PE=1 SV=1 - [BPL1_HUMAN]	48.13	6.47	1	3	3	3	2.664E6	726	80.7	5.62
P08107	Heat shock 70 kDa protein 1A/1B OS=Homo sapiens GN=HSPA1A PE=1 SV=5 - [HSP71_HUMAN]	43.54	6.08	1	3	3	3	2.623E6	641	70.0	5.66
Q9H4L5	Oxysterol-binding protein-related protein 3 OS=Homo sapiens GN=OSBPL3 PE=1 SV=1 - [OSBL3_HUMAN]	98.58	2.82	1	2	2	2	2.594E6	887	101.2	6.87
Q96H55	Unconventional myosin-XIX OS=Homo sapiens GN=MYO19 PE=2 SV=2 - [MYO19_HUMAN]	146.02	7.11	1	4	4	4	2.506E6	970	109.1	7.71
Q96FC9	Probable ATP-dependent DNA helicase DDX11 OS=Homo sapiens GN=DDX11 PE=1 SV=1 - [DDX11_HUMAN]	81.61	5.46	1	4	4	4	2.500E6	970	108.2	7.31
Q96RL7	Vacuolar protein sorting-associated protein 13A OS=Homo sapiens GN=VPS13A PE=1 SV=2 - [VP13A_HUMAN]	32.94	0.98	1	2	2	2	2.220E6	3174	360.0	6.33
Q3V6T2	Girdin OS=Homo sapiens GN=CCDC88A PE=1 SV=2 - [GRDN_HUMAN]	39.37	1.28	1	2	2	2	1.939E6	1871	215.9	6.21
Q5T447	E3 ubiquitin-protein ligase HECTD3 OS=Homo sapiens GN=HECTD3 PE=1 SV=1 - [HECD3_HUMAN]	33.58	3.25	1	2	2	2	1.510E6	861	97.1	5.64
Q5T4S7	E3 ubiquitin-protein ligase UBR4 OS=Homo sapiens GN=UBR4 PE=1 SV=1 - [UBR4_HUMAN]	29.07	0.37	1	2	2	2	1.214E6	5183	573.5	6.04

Uncropped blot images

Fig. 1F

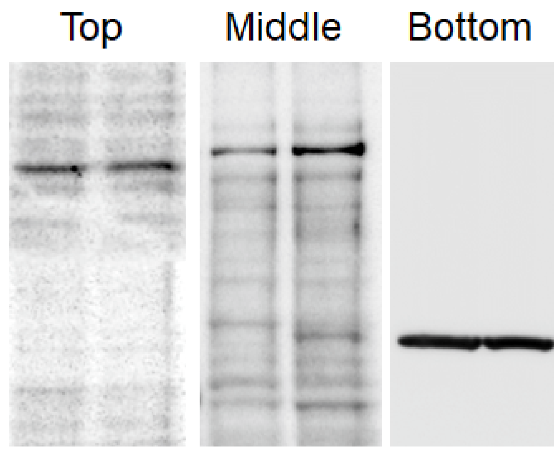

Fig. 1G

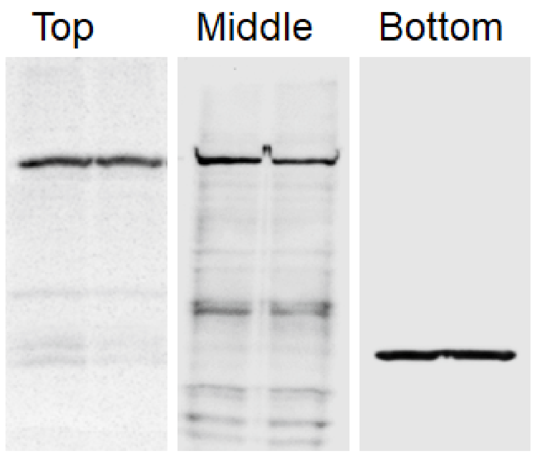

Fig. 1J

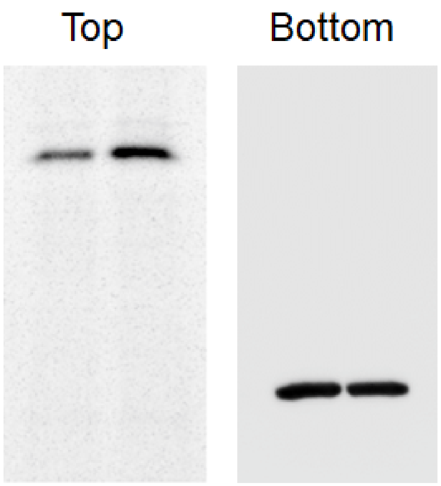

Fig. 1K

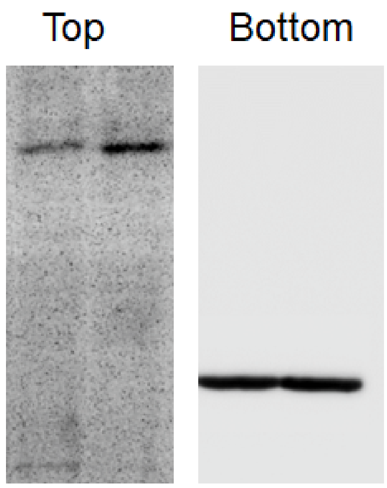

Fig. 2B

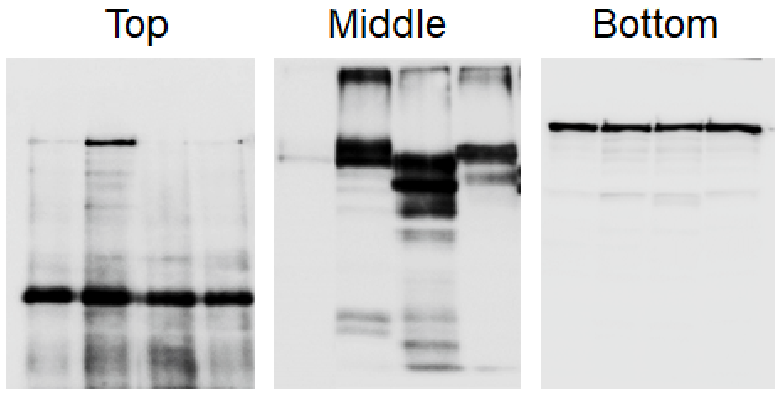

Fig. 2C

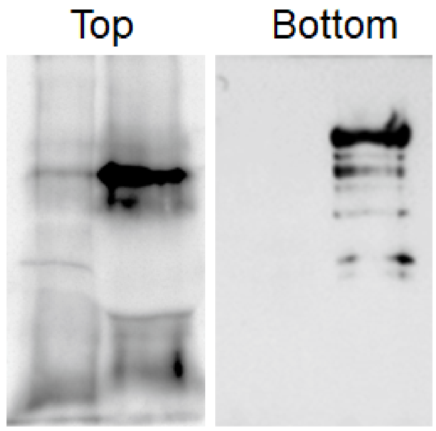

Fig. 3A

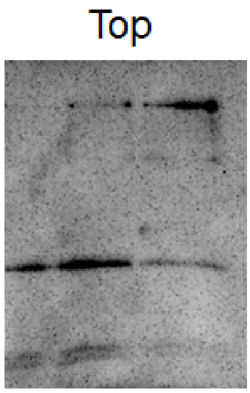

Fig. 3B

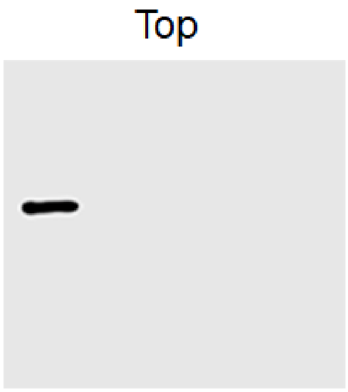

Fig. 3C

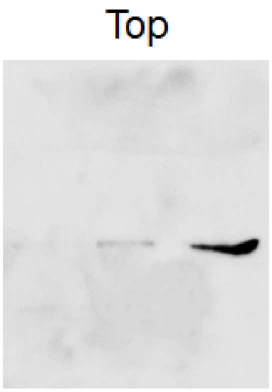

Fig. 3D

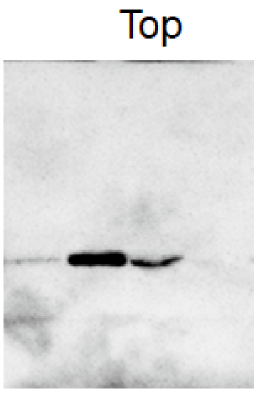

Fig. 4A

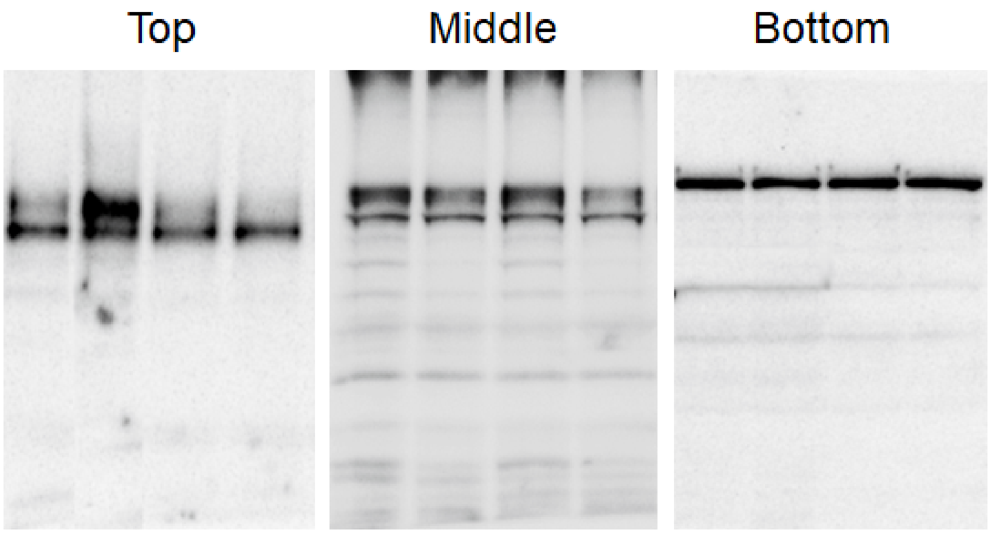

Fig. 4B

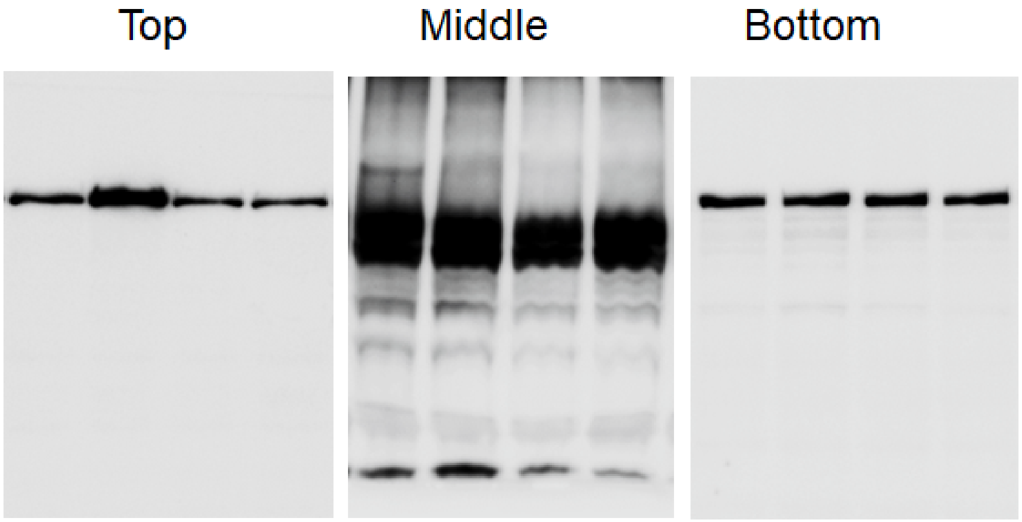

Fig. 4C

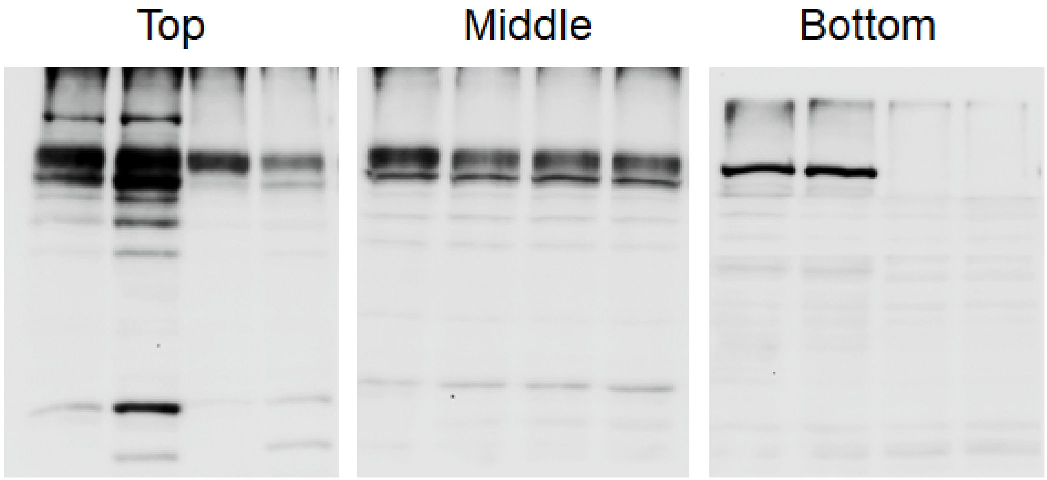

Fig. 4D

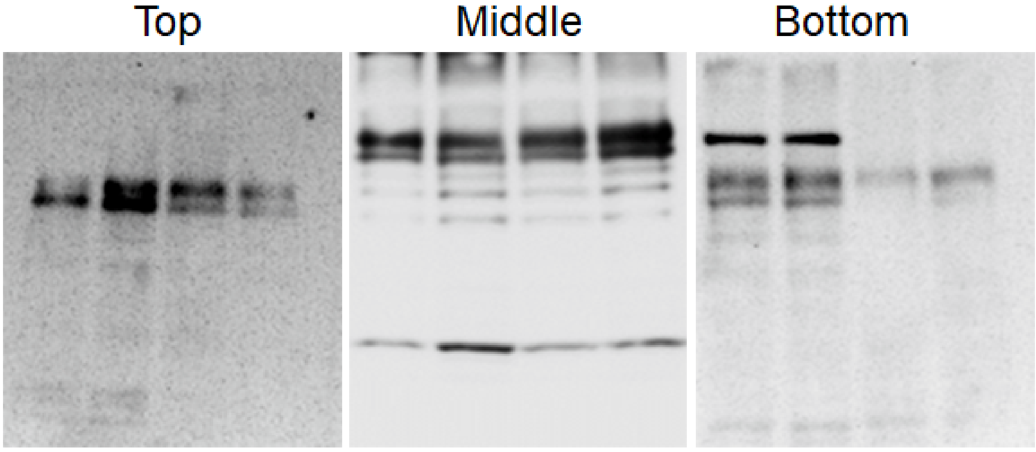

Fig. 4E

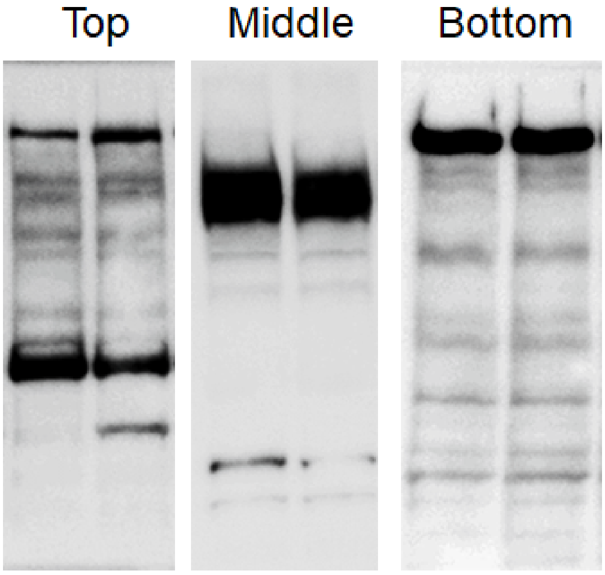

Fig. 4J

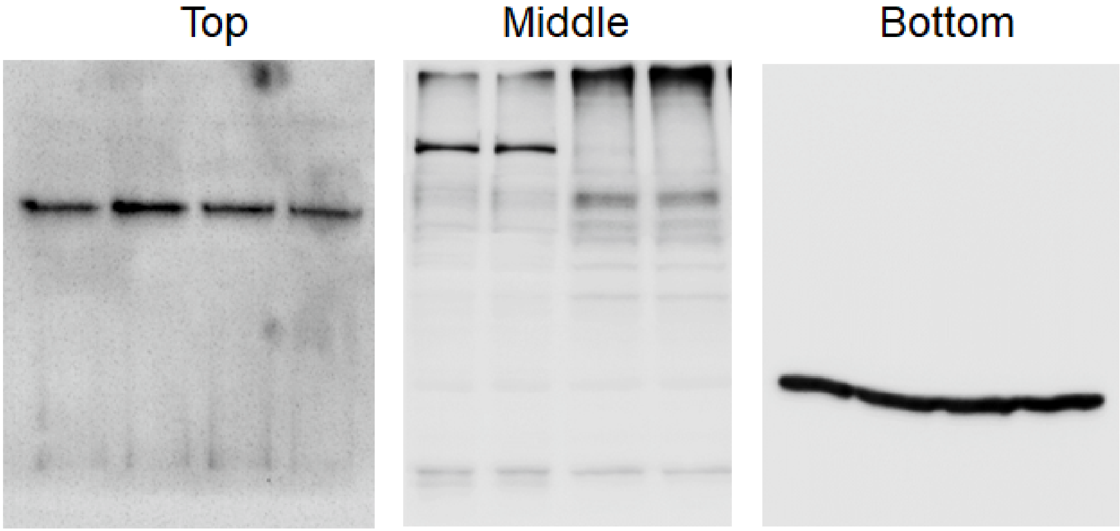

Fig. 5A

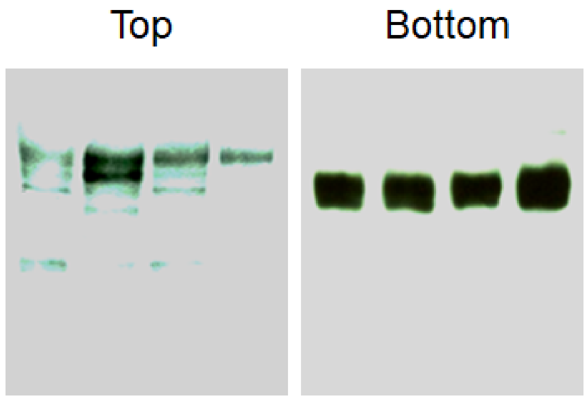

Fig. 5B

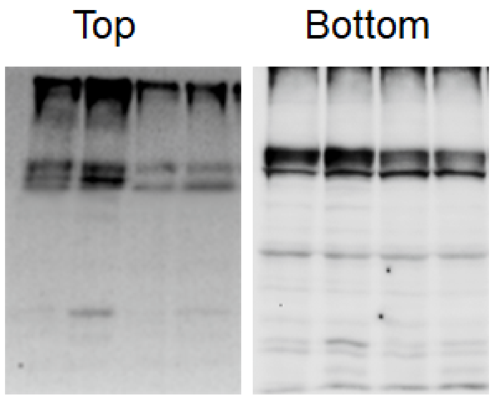

Fig. 5C

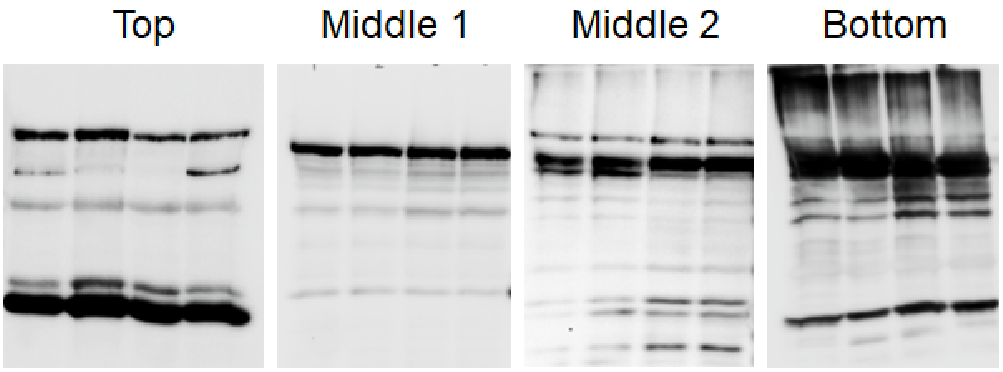

Fig. 5D

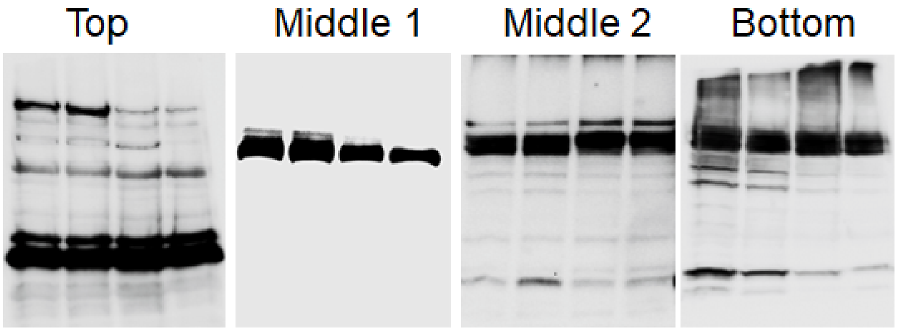

Fig. 5E

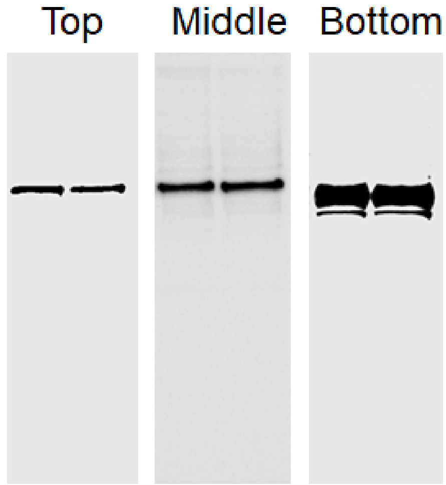

Fig. 5F

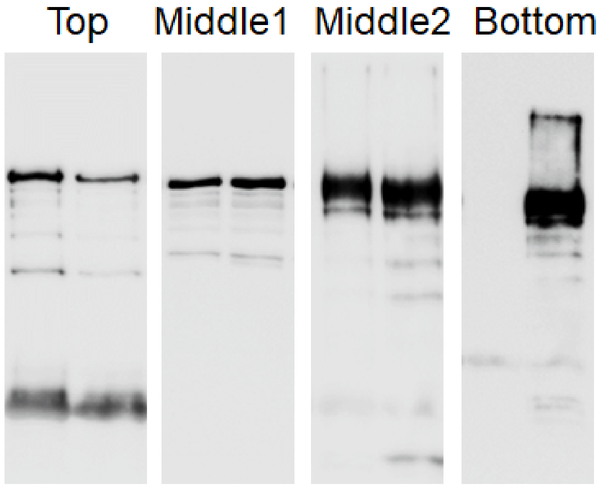

Fig. 5G

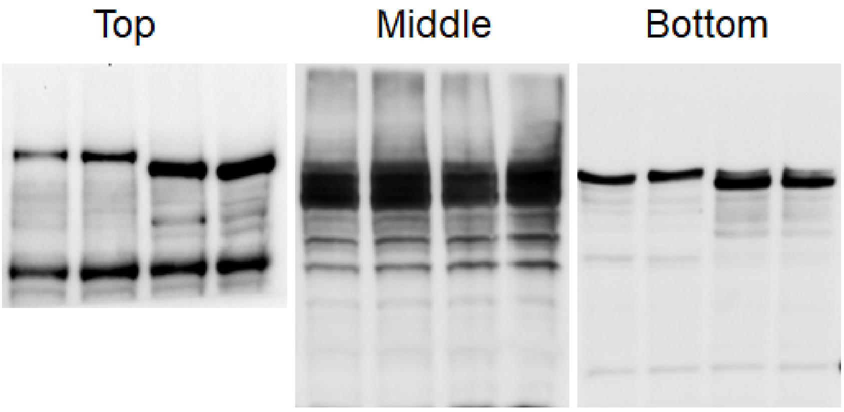

Fig. 5H

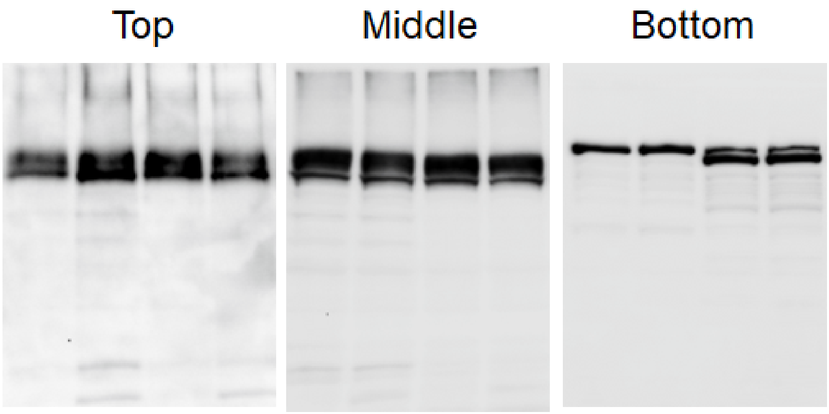

Fig. 5I

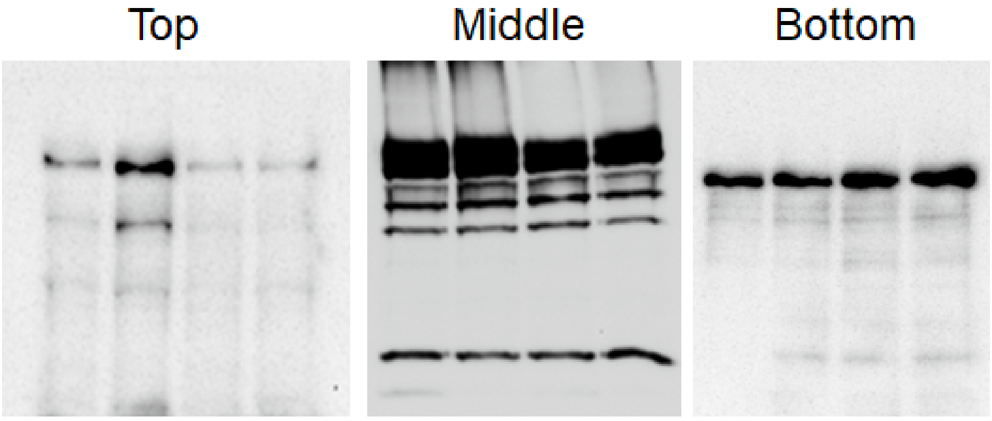

Fig. 5J

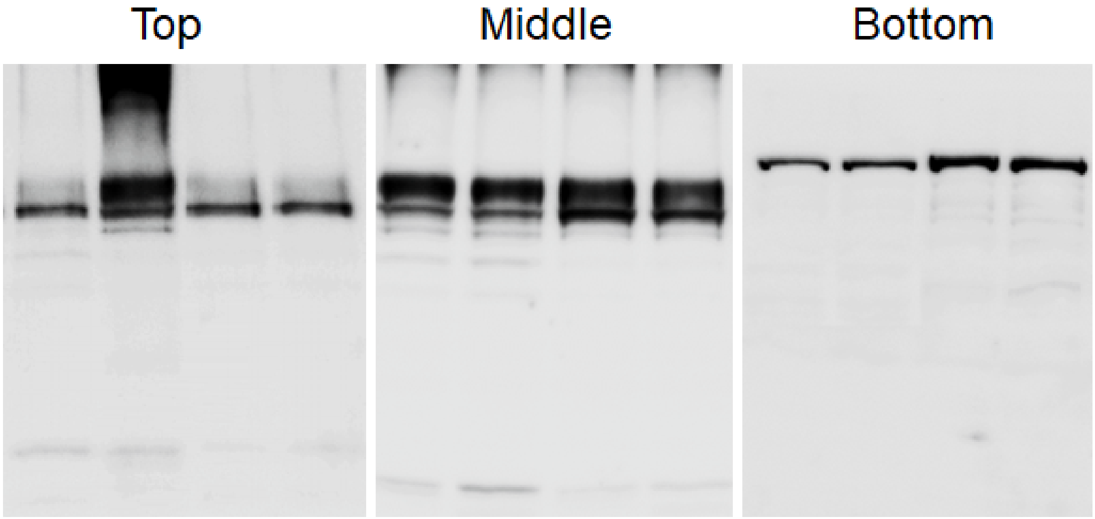

Fig. 5K

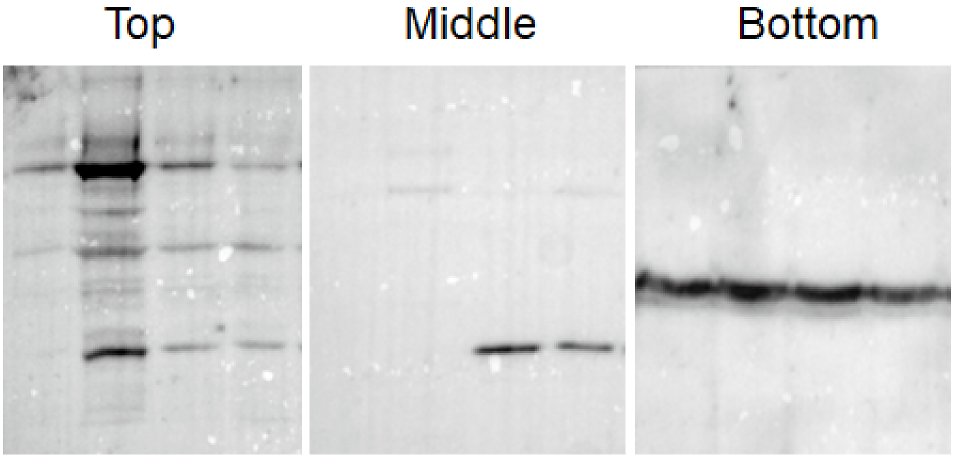

Fig. 5L

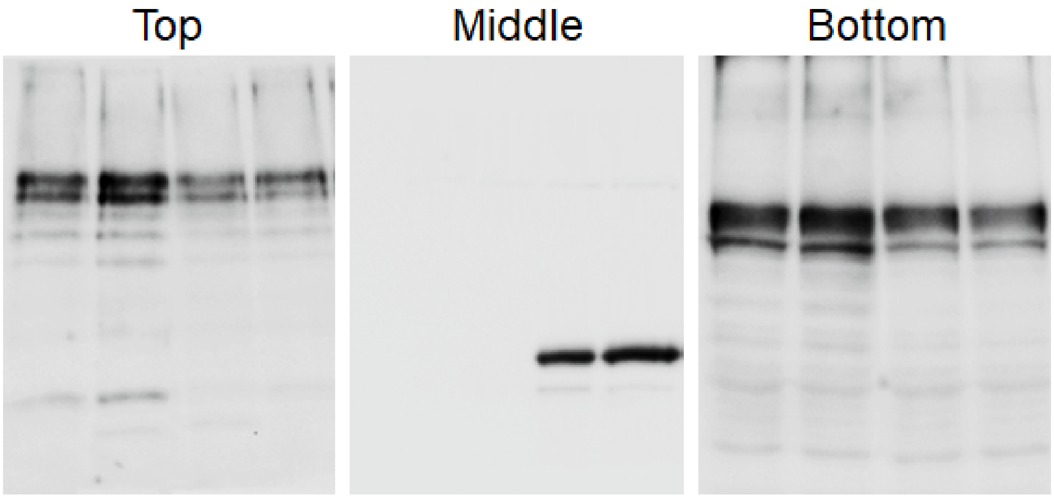

Fig. 8B

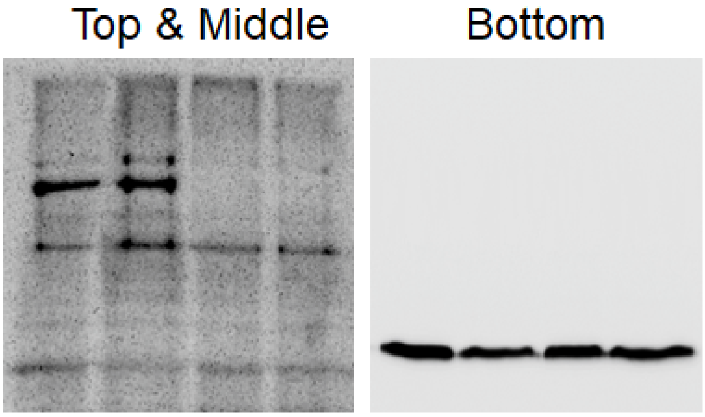

Fig. 8C

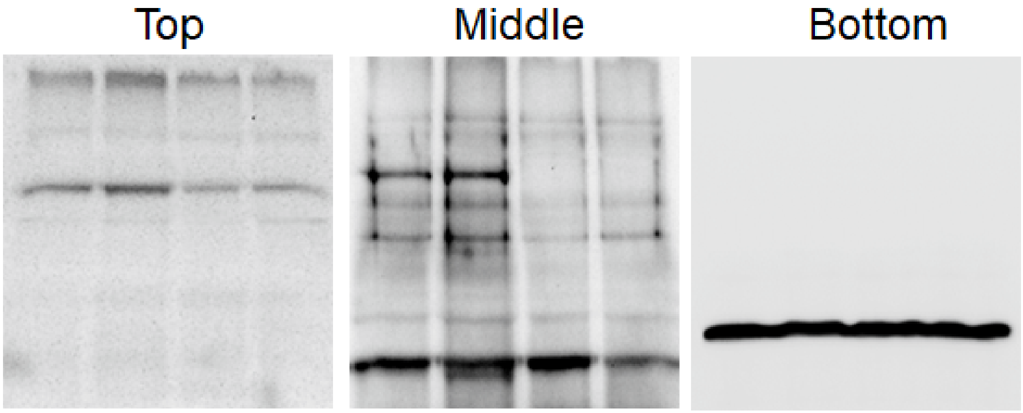

Supplementary Fig. 3A

Top

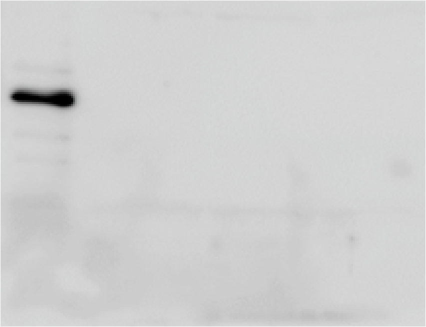

Supplementary Fig. 3C

Top

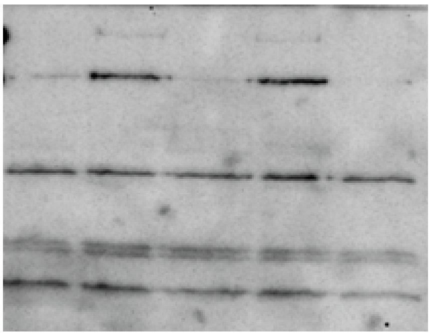

Supplementary Fig. 4A

Top Middle 1 Middle 2 Bottom

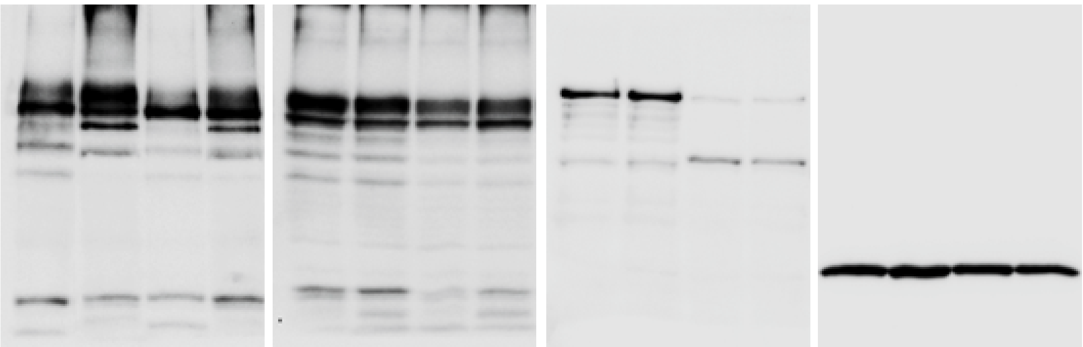

Supplementary Fig. 4C

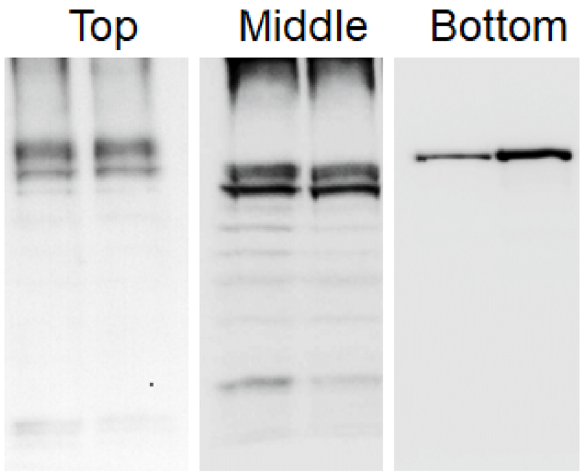

Supplementary Fig. 4D

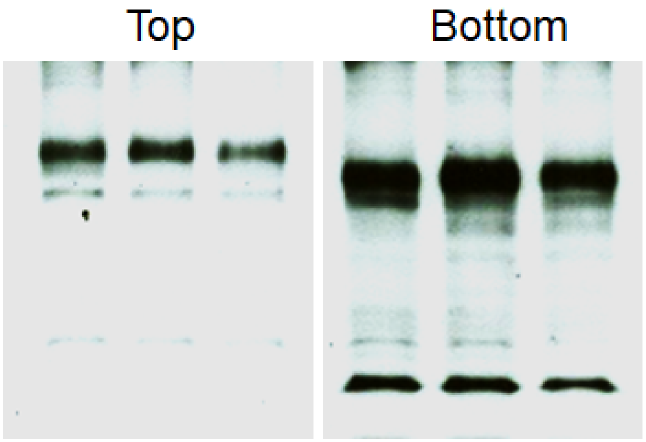

Supplementary Fig. 4E

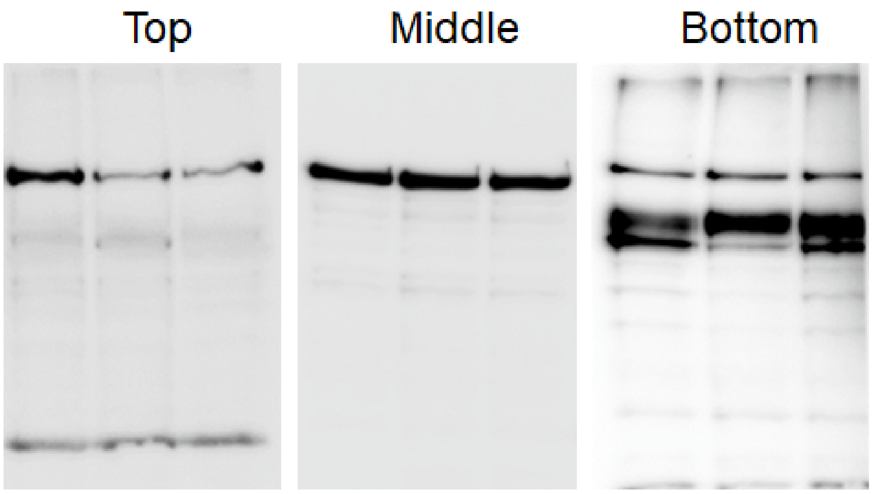

Supplementary Fig. 4F

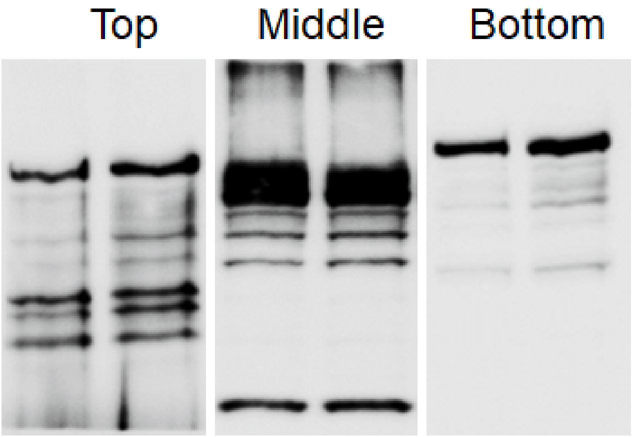

Supplementary Fig. 4G

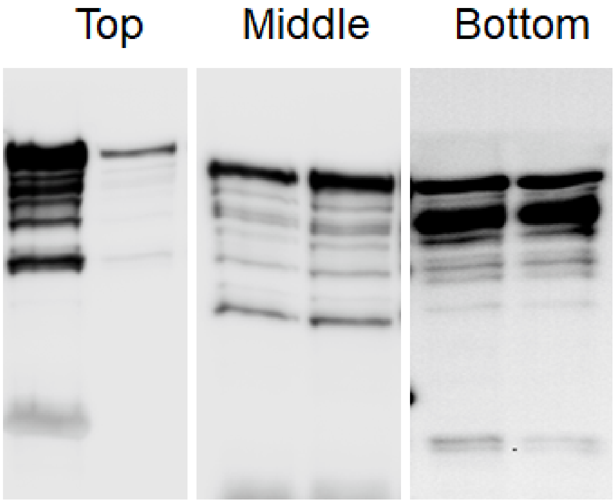

Supplementary Fig. 4H

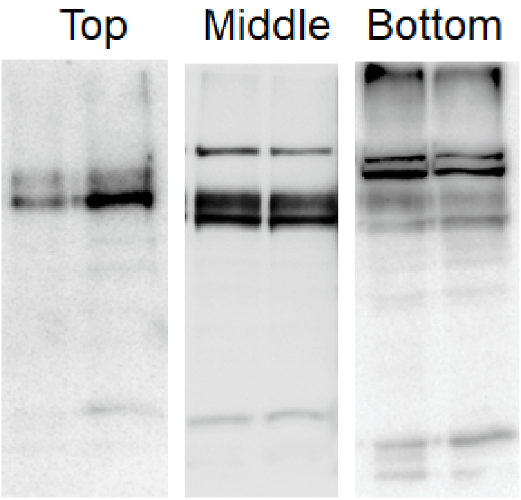

Supplementary Fig. 7A

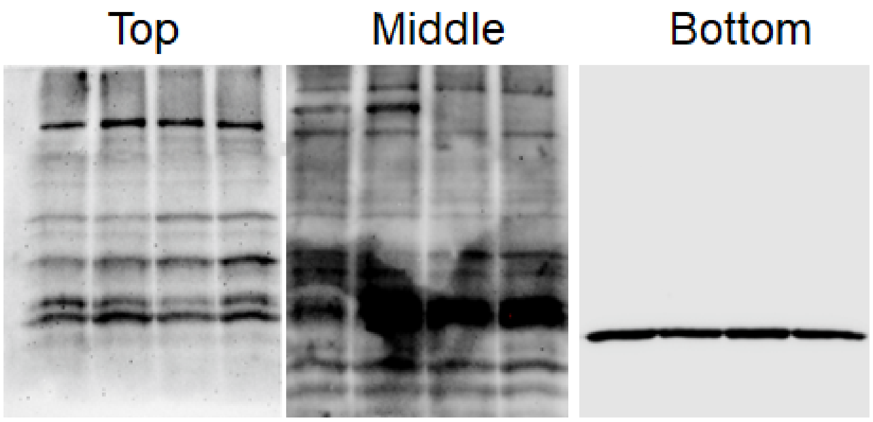

Supplementary Fig. 7B

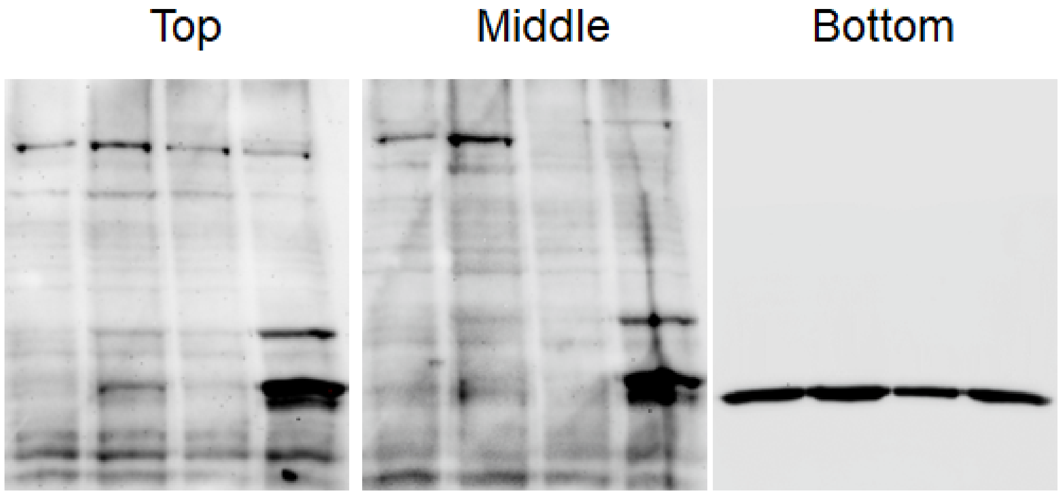

Supplement: awac462_Supplementary_Data [file awac462_supplementary_data.pdf]
